# Chain-length-selective cooperative assembly underlies β-glucan recognition by Dectin-1

**DOI:** 10.1038/s42004-026-02217-x

**Published:** 2026-11-10

**Authors:** Noriyoshi Manabe, Atsuji Kodama, Tokito Osaki, Hanako Chiba, Yuki Yamaguchi, Feng-Yueh Chan, Koichi Kato, Takayuki Uchihashi, Susumu Uchiyama, Yoshiki Yamaguchi

**Affiliations:** 1 Division of Structural Glycobiology, Institute of Molecular Biomembrane and Glycobiology, Tohoku Medical and Pharmaceutical University, Sendai, Japan; 2 Exploratory Research Center on Life and Living Systems (ExCELLS), National Institutes of Natural Sciences, Okazaki, Japan; 3 Graduate School of Engineering, The University of Osaka, Suita, Japan; 4 Graduate School of Science, Nagoya University, Nagoya, Japan; 5 Quantum-Based Frontier Research Hub for Industry Development (Q-BReD), Nagoya University, Nagoya, Japan; 6 Institute for Glyco-core Research (iGCORE), Nagoya University, Nagoya, Japan

**Keywords:** Glycobiology, Biophysical chemistry

## Abstract

Lectin receptors recognize specific glycan features within heterogeneous glycan populations to initiate immune signaling, yet how defined signals are extracted from structurally diverse ligand mixtures remains poorly understood. Here we report the molecular basis by which the innate immune receptor Dectin-1 recognizes the soluble β-glucan laminarin. Native mass spectrometry revealed that laminarin exists as heterogeneous chains spanning a degree of polymerization (DP) of 22–44, while Dectin-1 is strictly monomeric in solution. Isotopic mass tagging with uniformly ¹³C-labeled Dectin-1 established a preferred 3:3 receptor-to-laminarin stoichiometry (~80–85 kDa). Flow-induced dispersion analysis demonstrated cooperative binding with an apparent dissociation constant of 46 μM and a Hill coefficient of ~3, accompanied by a large increase in hydrodynamic radius. Analysis of the bound ligand fraction revealed selective enrichment of chains with DP 25–36, defining a preferred chain-length window. High-speed atomic force microscopy showed that individual Dectin-1 molecules undergo cooperative clustering on both laminarin and the longer β-glucan scleroglucan, while chemical crosslinking provided independent evidence for ligand-induced receptor association. Together, these findings demonstrate that β-glucan recognition by Dectin-1 is governed by chain-length selectivity and cooperative receptor assembly, consistent with a molecular ruler mechanism in which lectin receptors extract specific biological information from heterogeneous glycan populations.

## Abstract (web summary)

Lectin receptors on mammalian cell surfaces recognize specific glycan features within heterogeneous glycan populations to initiate immune signaling, yet how defined signals are extracted from structurally diverse ligand mixtures remains poorly understood. Here the authors report the molecular basis by which the innate immune receptor Dectin-1 recognizes the soluble β-glucan laminarin.

## Introduction

Glycans are intrinsically heterogeneous biomolecules. Unlike proteins or nucleic acids, glycan populations typically consist of mixtures of closely related structures that vary in chain length, branching, and substitution patterns. Despite this structural diversity, biological recognition often depends on specific glycan features. Establishing clear structure–activity relationships between defined glycan structures and their biological functions has proven difficult^1^, and relatively few studies have directly addressed how glycan heterogeneity influences receptor recognition and signaling^2,3^. Understanding how receptors extract specific biological information from heterogeneous glycan populations therefore represents a fundamental problem in glycobiology^4^.

Lectin receptors on mammalian cell surfaces play a central role in innate immunity by recognizing carbohydrate structures on pathogens and initiating downstream signaling cascades. Among these receptors, C-type lectins constitute the largest family and mediate Ca^2+^-dependent binding to diverse carbohydrate ligands^5–7^. Dectin-1, a C-type lectin-like receptor that specifically recognizes β-1,3-glucans abundant in fungal cell walls, is a key component of antifungal host defense^8–10^.

Engagement of Dectin-1 requires β-glucan oligosaccharides with a degree of polymerization (DP) of at least 10–11^11^ and is mediated by its extracellular lectin domain, in which residues such as Trp221 and His223 contribute critically to ligand binding^12^. As is typical for lectin–glycan interactions, one-to-one individual binding events are intrinsically weak^13^. Dectin-1 is thought to overcome this limitation through ligand-induced receptor oligomerization^14,15^, which increases avidity and facilitates productive signaling. In addition, β-glucans themselves are structurally heterogeneous, differing in backbone length, branching frequency, and side-chain composition; structural features that may strongly influence receptor recognition and higher-order assembly^16^. Indeed, studies have shown that β-glucan backbone chain length and side-chain branching affect Dectin-1 binding affinity over a markedly wide range (2.6 mM to 2.2 pM)^17^. However, the molecular basis of this chain-length dependence, including the binding stoichiometry, preferred glucan chain-length range, and role of cooperative receptor assembly, remains unresolved.

The β-glucan laminarin consists of a β-1,3-linked glucose backbone with periodic β-1,6-linked monoglucosyl branches approximately every seven residues^18^. It typically exhibits a DP of 20–30 and a molecular mass of ~4–6 kDa. Owing to its intrinsic heterogeneity, laminarin displays a broad mass distribution in mass spectrometry (MS)^18^. Previous studies have suggested that laminarin may induce oligomerization of Dectin-1, including tetramer formation^15^, and super-resolution imaging has identified β-glucan-induced Dectin-1 assemblies smaller than 15 nm that contain up to seven receptor molecules^19^. However, the precise binding stoichiometry, role of β-glucan chain length in receptor engagement, and extent of cooperative assembly in solution remain unresolved. Direct visualization of receptor–ligand dynamics at the single-molecule level might therefore provide critical insight into how β-glucan binding promotes receptor assembly.

Here we combined native and denaturing MS, flow-induced dispersion analysis (FIDA), high-speed atomic force microscopy (HS-AFM), and chemical crosslinking to elucidate the molecular basis of Dectin-1 recognition of β-glucans. By integrating complementary approaches capable of probing stoichiometry, cooperativity, dynamics, and receptor association, we sought to determine how Dectin-1 selectively recognizes heterogeneous β-glucan populations and converts weak carbohydrate interactions into stable receptor assemblies.

## Results and discussion

### Mass analysis of free unlabeled and ^13^C-labeled Dectin-1

**Fig. 1:**
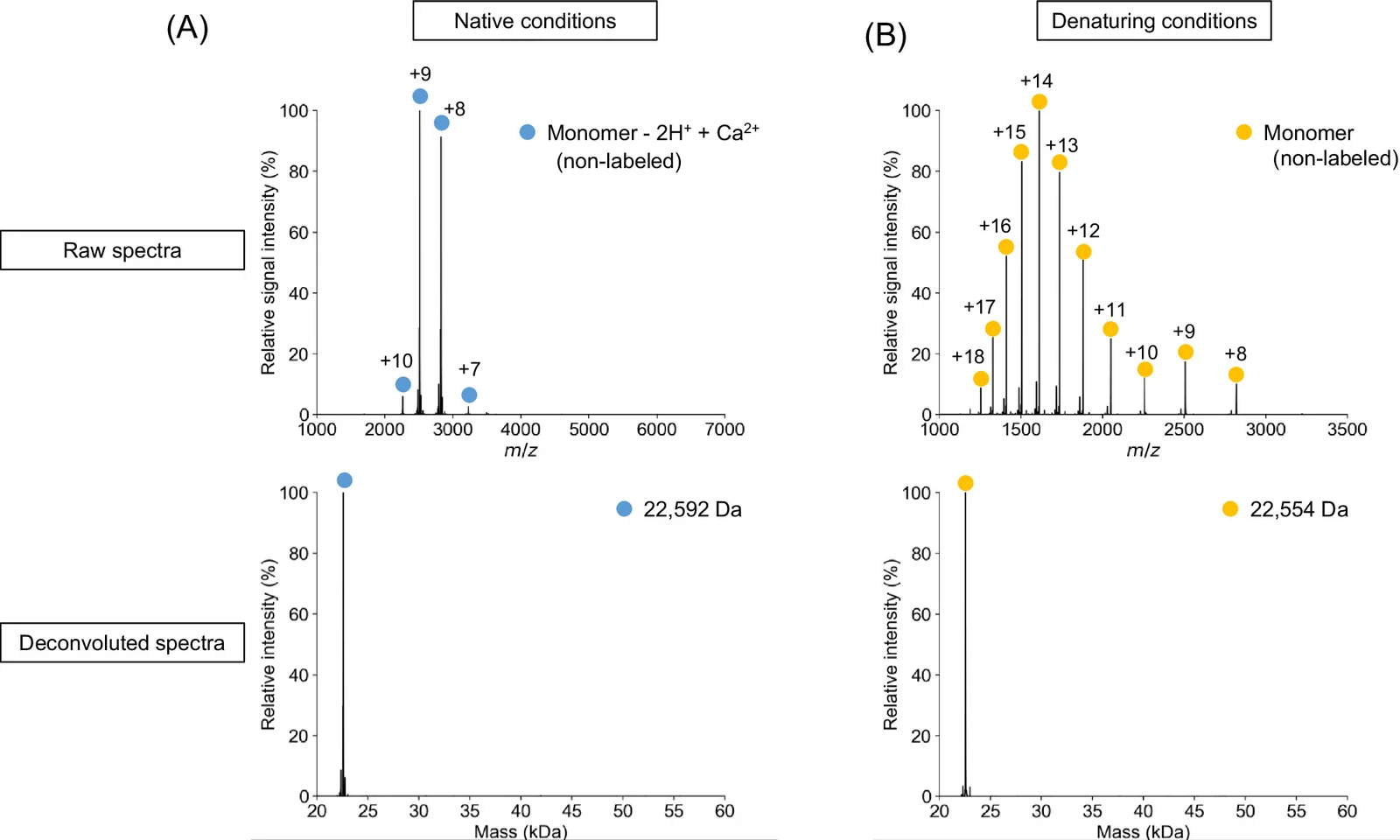
Monomeric state of unlabeled Dectin-1 analyzed by native and denaturing MS. **A** Mass spectra of unlabeled Dectin-1 under non-denaturing conditions, showing raw spectrum (top) and deconvoluted spectrum (bottom). **B** Mass spectra of unlabeled Dectin-1 under denaturing conditions, showing raw spectrum (top) and deconvoluted spectrum (bottom).

Native MS revealed that monomeric unlabeled Dectin-1 has a narrow charge-state distribution ranging from +8 to +10, consistent with a compact, folded conformation in solution (Fig. 1). In formic acid-denatured MS, by contrast, Dectin-1 had a broader charge-state distribution (+8 to +18), indicative of protein unfolding and increased solvent accessibility of ionizable residues.

Spectral deconvolution using UniDec yielded a molecular mass of 22,592 Da under native conditions and 22,554 Da under denaturing conditions. The latter value is in excellent agreement with the theoretical mass calculated from the amino acid sequence of Dectin-1 (22,554 Da), confirming the integrity of the recombinant protein. A mass increase of 38 Da was consistently observed under native conditions relative to denaturing conditions, suggesting the coordination of a single Ca^2+^ ion, as previously reported for the crystal structure of the Dectin-1 lectin domain^14^.

**Fig. 2:**
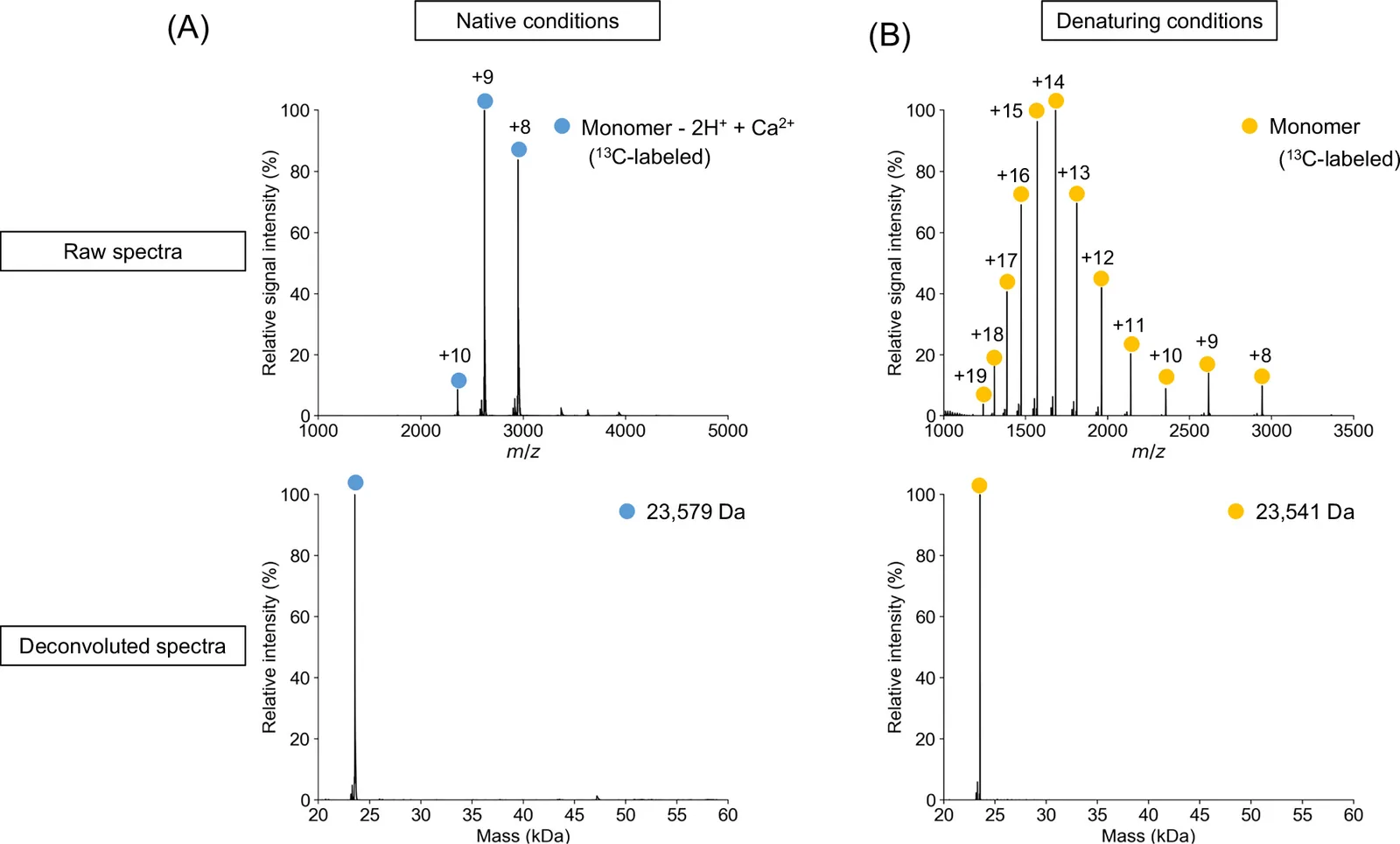
Monomeric state of ^13^C-labeled Dectin-1 analyzed by native and denaturing MS. **A** Mass spectra of ^13^C-labeled Dectin-1 under non-denaturing conditions, showing raw spectrum (top) and deconvoluted spectrum (bottom). **B** Mass spectra of ^13^C-labeled Dectin-1 under denaturing conditions, showing raw spectrum (top) and deconvoluted spectrum (bottom).

Native and denatured MS analyses were subsequently performed for uniformly ^13^C-labeled Dectin-1. Under native conditions, a molecular mass of 23,579 Da was observed, whereas denaturing conditions yielded 23,541 Da (Fig. 2). As with the unlabeled protein, the 38-Da mass difference between native and denatured spectra supports the presence of a single bound Ca²⁺ ion in the folded state. Based on the theoretical mass of unlabeled Dectin-1 (22,554 Da) and the experimentally determined mass difference upon ^13^C labeling, we estimated that 994 of the 1007 carbon atoms were replaced with a ^13^C isotope, corresponding to a ^13^C labeling efficiency of 98.7%. This value closely matches the nominal 99% isotope enrichment of the ^13^C_6_-glucose used as the sole carbon source during recombinant expression, indicating highly efficient incorporation of the labeled precursor.

### Mass analysis of free laminarin

**Fig. 3:**
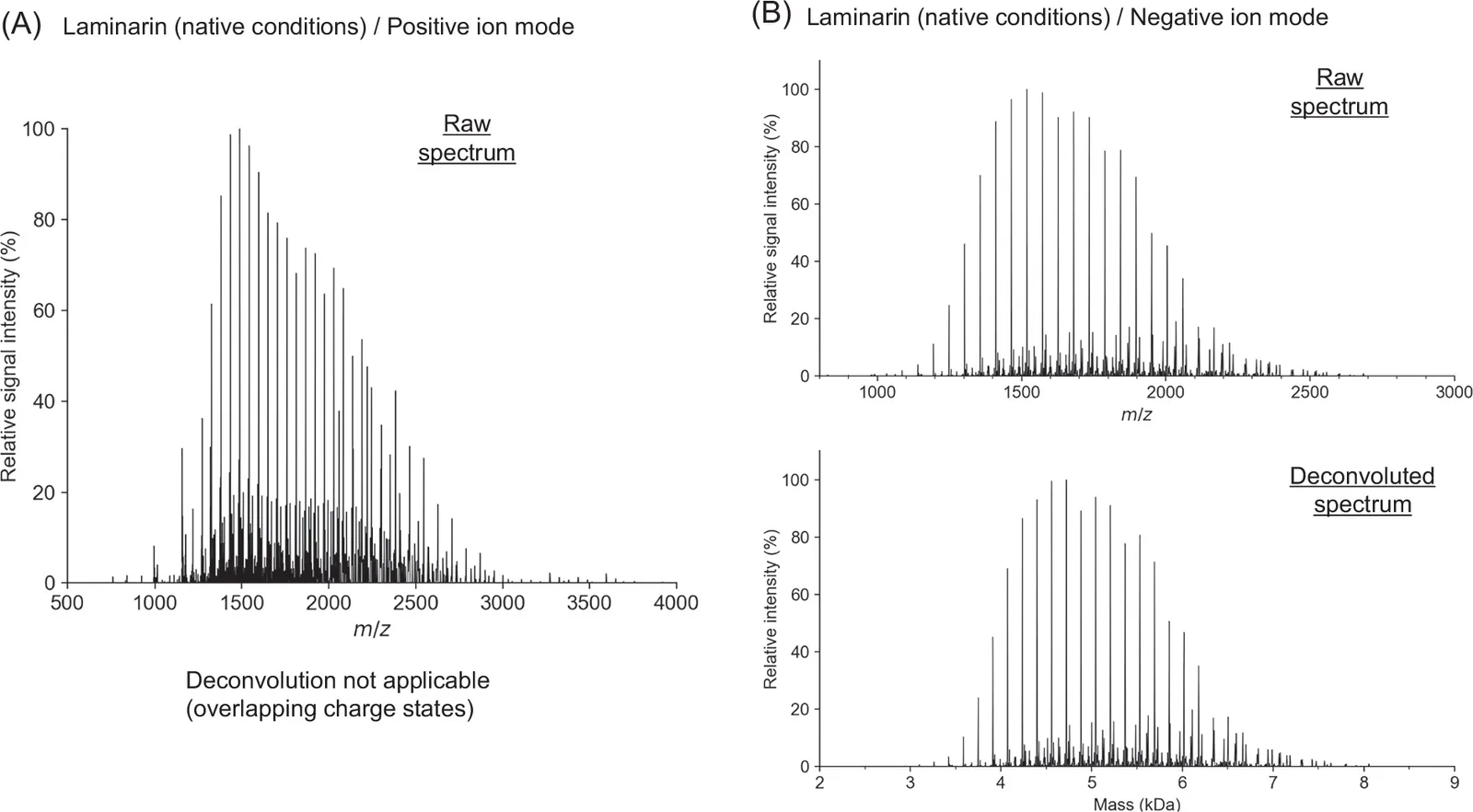
Laminarin heterogeneity revealed by native MS. **A** Raw mass spectrum of laminarin acquired in positive ion mode. The heterogeneous population of glucan chains has a degree of polymerization (DP) ranging from ~22 to 44, based on manual charge-state assignment (see text). **B** Mass spectra of laminarin acquired in negative ion mode, showing raw spectrum (top) and deconvoluted spectrum (bottom). The detected species span DP ~20–40, with adjacent peaks separated by ~162 Da, corresponding to a single hexose unit (C₆H₁₀O₅). The similar DP distributions observed in both ionization modes indicate that the heterogeneity reflects the intrinsic chain-length distribution of laminarin.

Laminarin is a β-(1→3) glucan with occasional β-(1→6) branching, resulting in intrinsic heterogeneity in chain length. To characterize the molecular mass distribution of free laminarin, we analyzed laminarin dissolved in ammonium acetate buffer by native MS (Fig. 3). In both positive and negative ion modes, the spectra displayed a series of well-resolved charge-state distributions corresponding to multiple polymeric species. Deconvolution using UniDec was successful for the spectrum obtained in negative ion mode (Fig. 3B), revealing a heterogeneous population with DP ranging from 20 to 40. In contrast, reliable deconvolution of the positive ion spectrum was not possible due to extensive charge-state overlap. However, detailed manual analysis of the raw spectrum, including assignment of the major charge-state series, indicated a comparable molecular mass distribution corresponding to a DP of 22–44 (Fig. 3A).

The mass difference between adjacent species was ~162 Da, corresponding to the mass of a single hexose unit (C_6_H_10_O_5_), confirming that the observed species represent glucan oligomers differing by one glucose residue. As similar DP distributions were observed in both ionization modes, the heterogeneity reflects the intrinsic chain-length distribution of laminarin rather than an ionization artifact. Therefore, because the native MS spectra of the Dectin-1–laminarin complexes were acquired in positive ion mode, we used the positive-ion spectra for subsequent analyses.

The intrinsic heterogeneity of laminarin provided an opportunity to examine potential chain-length selectivity in Dectin-1 recognition. To quantitatively describe the chain-length distribution, the number-average DP (${{DP}}_{n}$) and weight-average DP (${{DP}}_{w}$) were calculated from the deconvoluted peak intensities as follows:

$${{DP}}_{n}=\frac{\sum ({N}_{i}\cdot {{DP}}_{i})}{\sum {N}_{i}},\,{{\rm{and}}}\,{{DP}}_{w}=\frac{\sum ({N}_{i}\cdot {{DP}}_{i}^{2})}{\sum ({N}_{i}\cdot {{DP}}_{i})},$$

where ${N}_{i}$ represents the deconvoluted intensity corresponding to species with the degree of polymerization ${{DP}}_{i}$.

The calculated values were ${{DP}}_{n}$ = 30.3 and ${{DP}}_{w}$ = 31.3, yielding a polydispersity index (${{DP}}_{w}$/${{DP}}_{n}$) of 1.03^20^. This value indicates that laminarin has a relatively narrow chain-length distribution despite its natural origin^21^. The most abundant species in the distribution corresponded to a DP of 26; thus, this chain length predominates under the present preparation conditions.

Collectively, these results demonstrate that native MS facilitates direct and quantitative resolution of laminarin chain-length heterogeneity. The observed distribution provides a structural basis for interpreting Dectin-1 binding stoichiometry in subsequent complex analysis.

### Native MS analysis of Dectin-1–laminarin complexes

**Fig. 4:**
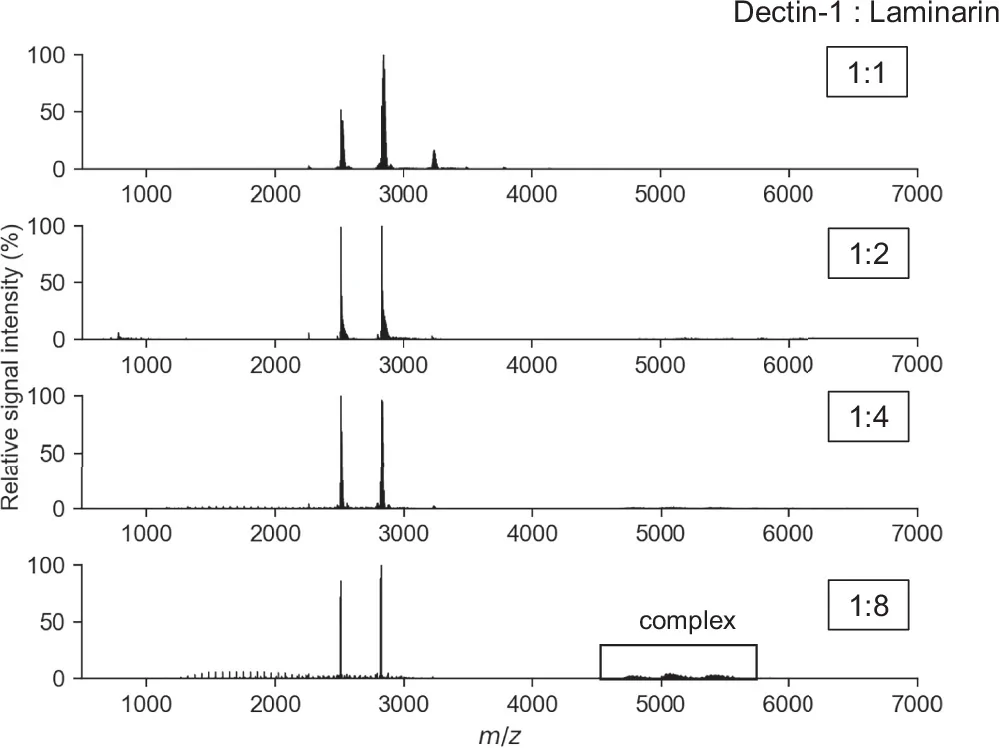
Optimization of the molar ratio for detecting Dectin-1–laminarin complexes by native MS. Mass spectra of a mixture of Dectin-1 and laminarin under non-denaturing conditions at a protein-to-laminarin molar ratio of 1:1, 1:2, 1:4, and 1:8. Increasing the laminarin concentration promoted the formation of Dectin-1–laminarin complexes, allowing identification of conditions optimal for complex detection.

To establish optimal conditions for detecting Dectin-1–laminarin complexes by native MS, spectra were initially acquired at protein-to-ligand molar ratios of 1:1, 1:2, 1:4, and 1:8. Among these conditions, a 1:8 ratio provided the most intense and well-resolved complex signals while minimizing excess unbound protein (Fig. 4). This ratio was therefore used for all subsequent analyses.

**Fig. 5:**
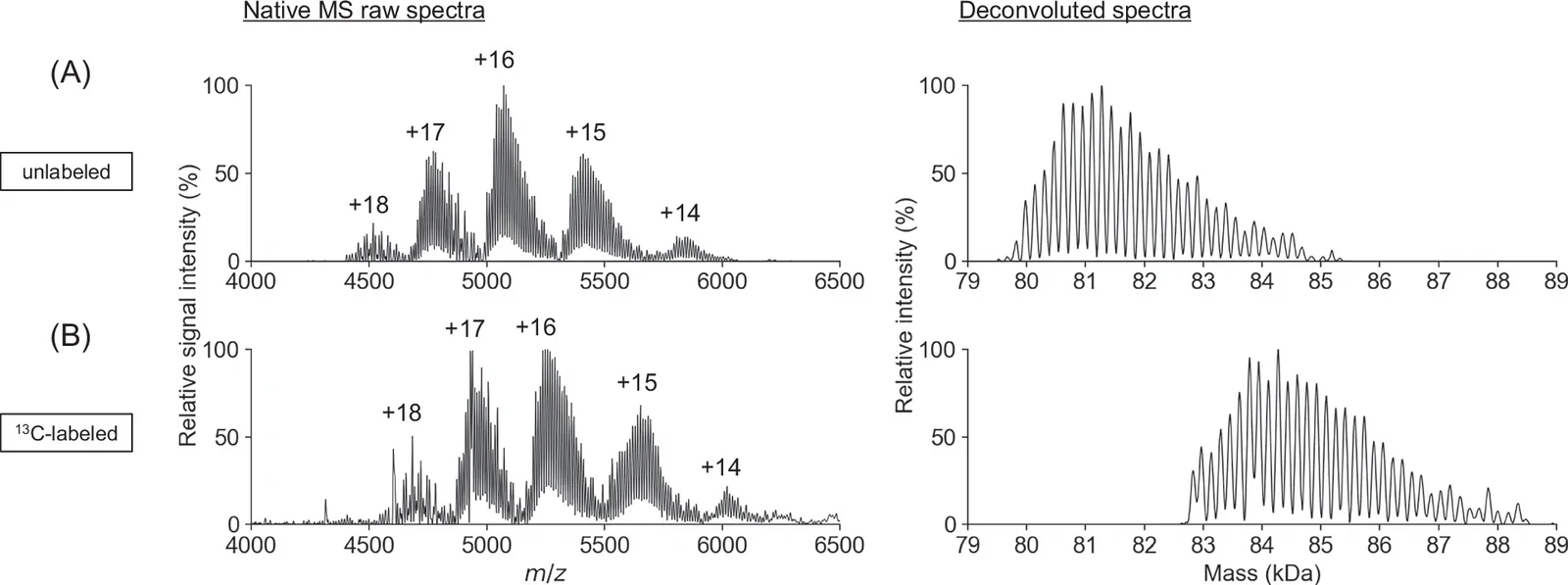
Native MS analysis of Dectin-1–laminarin complexes at a 1:8 molar ratio. **A** Mass spectra of laminarin bound to unlabeled Dectin-1 under non-denaturing conditions, showing raw spectrum (left) and deconvoluted spectrum (right). **B** Mass spectra of laminarin bound to ¹³C-labeled Dectin-1 under non-denaturing conditions, showing raw spectrum (left) and deconvoluted spectrum (right), facilitating independent verification of the complex mass.

Native MS of unlabeled Dectin-1 and laminarin (1:8 mixture) revealed peaks corresponding to free Dectin-1 monomer, free laminarin, and additional higher *m*/*z* species attributable to the putative complex (Fig. 5, upper left). Signals corresponding to the complex were primarily observed with charge states between +14 and +18, consistent with higher molecular weight assemblies. Notably, the broad distribution of these peaks paralleled the intrinsic heterogeneity of laminarin chain lengths, suggesting that complex formation occurs across multiple DP.

To resolve charge-state heterogeneity, spectra were deconvoluted using UniDec to convert *m*/*z* values into zero-charge molecular masses (Fig. 5, upper right). The resulting mass spectrum displayed a series of peaks separated by ~162 Da, corresponding to the mass of a single glucose residue (C_6_H_10_O_5_). In total, 32 discrete complex species were identified, reflecting incorporation of laminarin chains of varying lengths into the complex.

Equivalent experiments were performed using uniformly ^13^C-labeled Dectin-1. Native MS revealed complex-associated charge states between +14 and +18, similar to the unlabeled protein (Fig. 5, lower left). Deconvolution again yielded peaks spaced by ~162 Da, identifying 35 distinct complex species (Fig. 5, lower right) and confirming that isotopic labeling did not alter the overall binding pattern.

Direct comparison of unlabeled and ^13^C-labeled complexes revealed an overall mass difference of ~3000 Da. Based on our above finding that ^13^C labeling replaces 994 of the 1007 carbon atoms in each Dectin-1 monomer (increasing mass by ~994 Da per monomer), the observed ~3000 Da shift indicates the presence of three Dectin-1 molecules per complex (3 × 994 ≈ 2982 Da). This isotopic mass tagging thus provided unambiguous evidence that three Dectin-1 molecules are incorporated into the Dectin-1–laminarin complex.

Next, to determine laminarin stoichiometry, experimental complex masses were compared with theoretical models assuming combinations of 2–4 laminarin molecules per three Dectin-1 molecules. Our initial mass estimates allowed 2:3, 3:3, or 4:3 laminarin:Dectin-1 assemblies; however, precise fitting of the deconvoluted mass values excluded the 2:3 and 4:3 models due to the following systematic mass deviations. Under the assumption of a 2:3 stoichiometry (i.e., two glucan chains bound to three Dectin-1 molecules), the laminarin population would be dominated by longer chains corresponding to DP 37–54. Conversely, under a 4:3 stoichiometry, predominantly shorter laminarin chains within the DP 18–36 range would be required. In contrast, the experimentally observed distribution spanned DP 22–44. Thus, the laminarin size distribution, together with the native MS stoichiometry, supports the formation of a 3:3 laminarin:Dectin-1 complex.

**Fig. 6:**
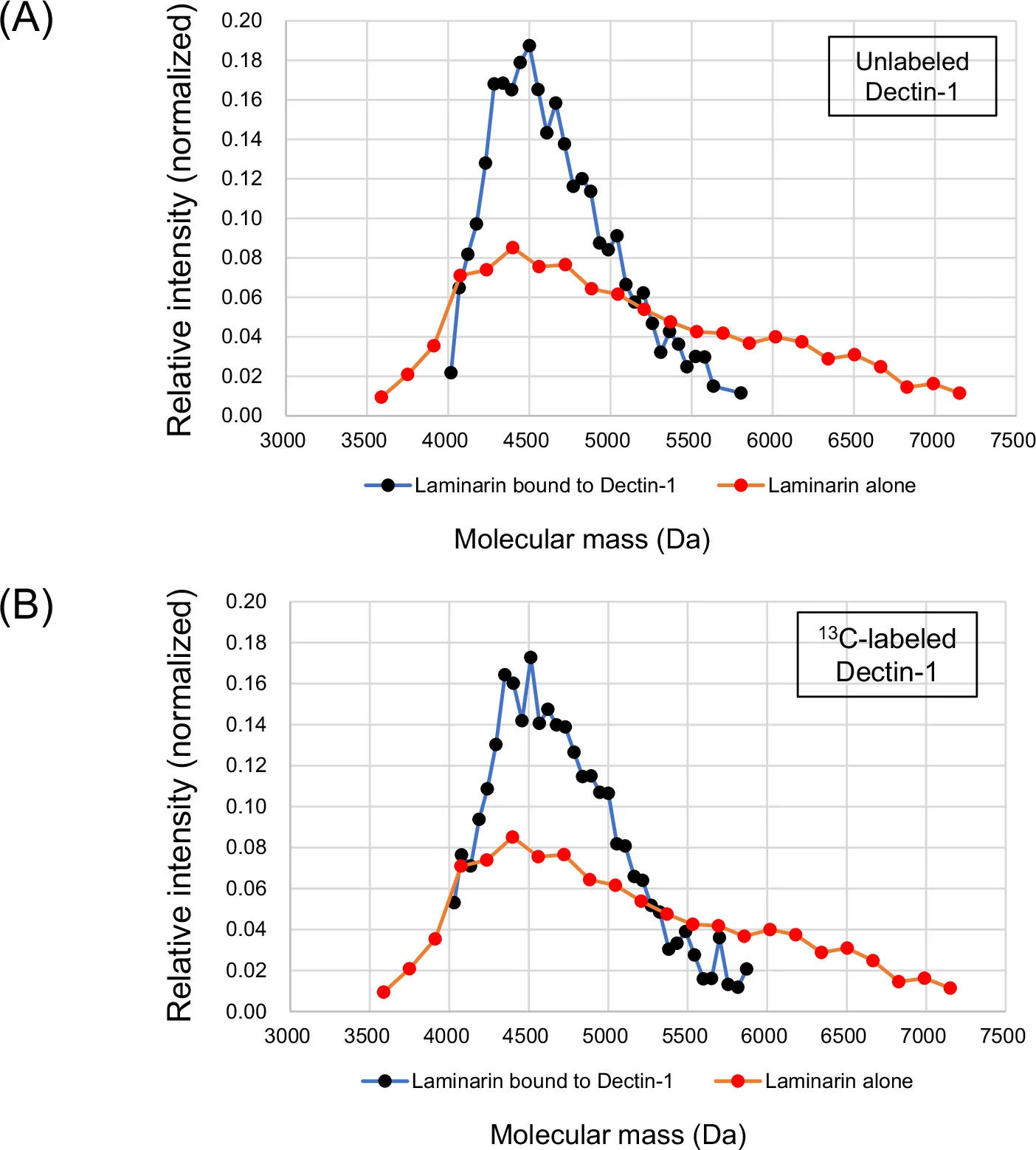
Comparison of the molecular mass distribution of laminarin alone and in complex with Dectin-1. **A** Molecular mass distribution of laminarin alone (orange) and bound to unlabeled Dectin-1 (blue). The molecular mass distribution of laminarin incorporated into the complex was calculated from the experimentally determined masses of the Dectin-1–laminarin complexes obtained by native MS. Assuming a 3:3 stoichiometry, the theoretical mass of three Dectin-1 molecules was subtracted from the total complex mass, and the remainder was divided by three, yielding the molecular mass distribution of individual laminarin chains bound to the complex. **B** Molecular mass distribution of laminarin alone (orange) and bound to ^13^C-labeled Dectin-1 (blue). The molecular mass distribution of laminarin incorporated into the complex was calculated as in (**A**). To aid comparison, each molecular mass distribution was normalized by setting the total area of the distribution to 1. Comparison of the distributions indicated that laminarin chains with a DP of ~25–36 are preferentially enriched in the bound fraction.

Assuming a 3:3 stoichiometry, we calculated the mass contribution of laminarin in each complex by subtracting the theoretical mass of three Dectin-1 molecules from the total complex mass and dividing the remainder by three. The resulting laminarin mass distribution was directly compared with that of free laminarin (Fig. 6). Whereas free laminarin exhibited a DP range of 22–44, laminarin incorporated into the complex was enriched for chains with DP 25–36. Longer chains (molecular mass >5000) were less frequently observed within the isolated 3:3 complex, suggesting that chains within this size range most efficiently support formation of the stoichiometry observed by native MS. Conversely, very short chains were also less frequently observed in the complex, implying that a minimum chain length might be required to stabilize the observed 3:3 Dectin-1–laminarin complex.

Taken together, these results demonstrate that native MS, combined with isotopic mass tagging, facilitates the direct determination of both protein and ligand stoichiometry in heterogeneous polysaccharide–receptor complexes, revealing selective enrichment of laminarin chains with DP 25–36 within the observed 3:3 Dectin-1–laminarin complex.

### FIDA reveals cooperative assembly of Dectin-1 in solution

**Fig. 7:**
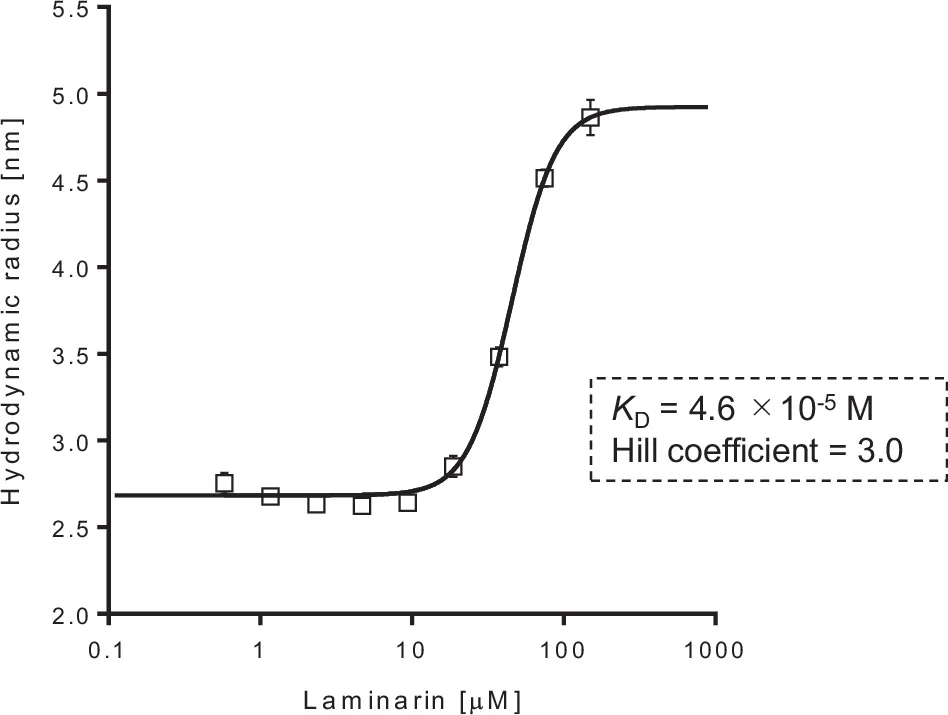
FIDA shows cooperative assembly of Dectin-1 upon laminarin binding. Hydrodynamic radius (*R*_*h*_) of Dectin-1 plotted as a function of laminarin concentration on a logarithmic scale. Increasing laminarin concentrations induced a progressive increase in *R*_*h*_ from 2.68 nm (free Dectin-1) to 4.92 nm under saturating conditions, indicating the formation of a higher-order complex in solution. Data were fitted by a non-linear regression model based on the Hill equation, which yielded an apparent dissociation constant (*K*_*D*_) of 46 μM and a Hill coefficient of ~3, indicating strong positive cooperativity. Data points represent mean ± S.D. (*n* = 3).

To quantify binding affinity and evaluate cooperative assembly in solution, we performed FIDA. Upon titration with laminarin, the hydrodynamic radius (*R*_*h*_) of Dectin-1 increased from 2.68 ± 0.02 nm (mean ± S.E.) in the free state to 4.92 ± 0.06 nm under saturating conditions (Fig. 7), indicating the formation of a higher-order assembly in solution. The observed *R*_*h*_ of free Dectin-1 (2.68 nm) agrees well with the hydrodynamic radius calculated from the crystal structure of the mouse Dectin-1 lectin domain (2.75 nm; PDB ID, 2BPD)^14^. Without a high-resolution structure of the Dectin-1–laminarin complex, it is not possible to calculate the expected *R*_*h*_ of the assembled complex directly; nevertheless, the substantial increase in *R*_*h*_ observed in the presence of laminarin is consistent with the incorporation of multiple Dectin-1 molecules into a higher-order receptor–glucan complex. Together with the 3:3 stoichiometry observed by native MS, these data are consistent with a model in which laminarin promotes cooperative assembly of Dectin-1 into the 3:3 receptor–glucan complex.

To quantify the interaction, binding curves were fitted by using a Hill model^22,23^, which yielded an apparent dissociation constant (${K}_{D}$) of 46 μM and a Hill coefficient (n) of 3.0, indicating strong positive cooperativity in laminarin binding. These values are consistent with our previous analysis using intrinsic tryptophan fluorescence of Dectin-1, which determined the *K*_*D*_ and Hill coefficient to be 39 μM and 3.1, respectively^15^. Although the Hill coefficient does not directly define stoichiometry, a value close to 3 suggests that laminarin binding involves highly cooperative multivalent interactions.

Importantly, the observed magnitude of the hydrodynamic expansion is consistent with assembly of multiple Dectin-1 molecules. Because hydrodynamic radius scales with the cube root of molecular mass and is strongly influenced by molecular shape, *R*_*h*_ measurements alone cannot unambiguously distinguish between 3:3 and higher-order assemblies. However, our native MS data demonstrated a precise +3000 Da mass increase upon ^13^C labeling, corresponding to the incorporation of three Dectin-1 molecules in the complex. The FIDA-derived cooperativity therefore provides independent evidence that complex formation involves concerted multivalent interactions, complementing the 3:3 stoichiometry determined by native MS.

Previous studies have suggested that higher-order complexes form between Dectin-1 and laminarin. For example, dynamic light scattering and analytical ultracentrifugation indicated that Dectin-1 forms oligomers ranging from dimers to tetramers^14^. In addition, size exclusion chromatography coupled with multi-angle light scattering suggested a 4:4 stoichiometry for the Dectin-1–laminarin complex^15^. In contrast, our direct mass spectrometric analysis established a predominant 3:3 Dectin-1–laminarin assembly. This discrepancy may arise from different experimental conditions, including variations in the laminarin-to-protein ratio, lot-to-lot differences in the laminarin DP distribution, or uncertainties inherent in the analytical models used in previous studies. In particular, the estimation of molecular stoichiometry in light-scattering analyses depends on assumptions regarding molecular shape and the differential refractive index increment (*dn*/*dc*), which may introduce systematic errors^15^.

Together, these data support a model in which laminarin engagement promotes cooperative association of multiple Dectin-1 molecules and stabilizes higher-order receptor–glucan complexes in solution. The pronounced cooperativity suggests that ligand binding is not independent across receptors but instead occurs through concerted multivalent interactions, which probably enhance avidity and stabilize receptor clustering. Such cooperative assembly provides a mechanistic basis for how Dectin-1 selectively recognizes heterogeneous β-glucans and converts weak monovalent interactions into stable signaling-competent complexes.

### High-speed AFM observation of Dectin-1–laminarin complex formation

Next, we used HS-AFM to directly visualize the interaction between Dectin-1 and laminarin under near-physiological conditions. As a control, we first observed Dectin-1 alone (1 μM) deposited onto a mica surface in PBS (pH 7.4) (Movie S1; Fig. S1). Although a few stationary bright spots were occasionally observed, most Dectin-1 molecules diffused rapidly across the mica surface, precluding clear identification of their shape. This behavior showed that Dectin-1 adsorbs only weakly to the mica substrate.

**Fig. 8:**
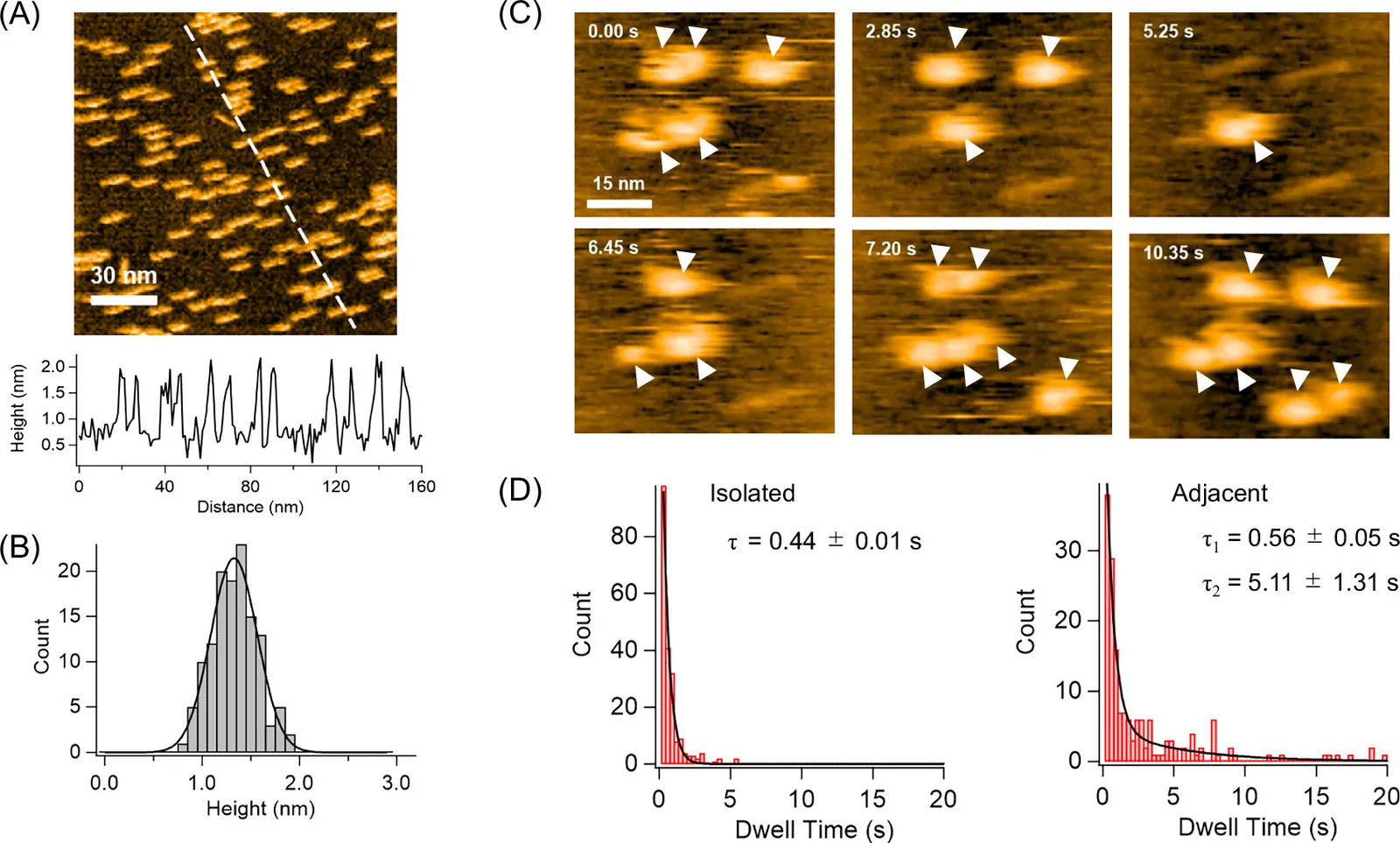
High-speed AFM visualization of laminarin and its interaction with Dectin-1. **A** HS-AFM image of laminarin (100 μM) adsorbed on mica in PBS (pH 7.4); laminarin appears as distinct rod-like structures. The height profile along the dashed line is shown below. **B** Histogram of rod heights (*n* = 128). The unbroken line represents a Gaussian fit (peak, 1.33 ± 0.01 nm; *σ* = 0.34 ± 0.01 nm). **C** Sequential HS-AFM images recorded after addition of Dectin-1 (0.3 μM) to laminarin (100 μM) pre-adsorbed on mica in PBS (pH 7.4). Imaging speed, 0.15 s/frame. Arrowheads indicate Dectin-1 molecules repeatedly binding to and dissociating from laminarin chains. **D** Dwell time histograms of Dectin-1 on laminarin for isolated molecules (*n* = 209) and molecules adjacent to at least one other Dectin-1 on the same chain (*n* = 169). Unbroken line represents fit by a single-exponential decay (isolated, *τ* = 0.44 ± 0.01 s) and a double-exponential decay (adjacent, *τ*_1_ = 0.56 ± 0.05 s; *τ*_2_ = 5.11 ± 1.31 s), respectively.

When laminarin was deposited onto mica at 100 μM, by contrast, distinct rod-like structures were observed (Fig. 8A). Analysis of the rod length distribution by Gaussian fitting (Fig. S2A) gave a peak position of 9.96 ± 0.05 nm. Assuming an axial rise per residue of 0.29 nm for β-1,3-glucan^16,24^, this length corresponds to a DP of ~25–36, coinciding with the laminarin chain lengths preferentially incorporated into the 3:3 Dectin-1–laminarin complex determined by native MS. Interestingly, the rod-like structures were observed to align uniformly on the mica surface, with rods on adjacent terraces showing orientations differing by ~60° (Fig. S2B), possibly reflecting epitaxial adsorption of laminarin in register with the crystalline lattice of the mica surface.

The heights of the rod-like structures were nearly uniform (Fig. 8A), and Gaussian fitting of the height distribution (Fig. 8B) gave a peak position of 1.33 ± 0.01 nm (*σ* = 0.34 ± 0.01 nm). To assign this height, we generated a simulated AFM image from the model structure of a β-glucan triple helix using a collision simulation (tip radius, 0.5 nm; cone angle, 10°) (Fig. S2C). The simulated height of the helix was ~1.2 nm, which agrees well with the experimentally observed rod height (~1.3 nm). In addition, simulated AFM images generated from a single-helical model exhibited pronounced periodic surface features that were not apparent in the experimental images (Fig. S2D), whereas the triple-helical model reproduced the observed smooth rod-like morphology more closely. Although HS-AFM alone cannot unambiguously determine glucan conformation, these observations indicate that the laminarin structures adsorbed on mica are more consistent with a triple-helical assembly than with an isolated single-chain helix.

It should be noted that the higher-order structures of β-1,3-glucans are strongly influenced by chain length, branching pattern, concentration, and solvent conditions. β-Glucans have been reported to form multi-chain helical assemblies, but the precise supramolecular organization of laminarin remains incompletely understood and may depend on experimental conditions^25–27^. Therefore, while our AFM observations are consistent with a triple-helical assembly, additional structural studies will be required to establish the molecular architecture of laminarin under native solution conditions.

Upon addition of Dectin-1 to the observation buffer (final concentration 0.3 μM), blinking bright spots appeared on the pre-adsorbed laminarin rods, and their number gradually increased over time (Movie S2; Fig. S3). More detailed observations revealed that Dectin-1 did not bind statically to laminarin but instead exhibited dynamic association–dissociation behavior (Movie S3; Fig. 8C). As an example, Fig. 8C shows four laminarin chains adsorbed on the mica surface, with Dectin-1 molecules (arrowheads) repeatedly binding to and dissociating from these chains.

Strikingly, the dwell time of Dectin-1 on laminarin differed markedly depending on whether the molecule was isolated or adjacent to one or more Dectin-1 molecules on the same laminarin chain during the dwell period (Fig. 8D). The dwell time distribution of isolated single molecules was well described by a single-exponential decay, yielding a lifetime of *τ* = 0.44 ± 0.01 s. In contrast, when Dectin-1 molecules were adjacent to at least one neighboring molecule on the same chain, the dwell time distribution was best fitted by a double-exponential decay, revealing two kinetically distinct populations: a short-lived component (*τ*_1_ = 0.56 ± 0.05 s) and a long-lived component (*τ*_2_ = 5.11 ± 1.31 s). The value of *τ*_2_ was ~10-fold greater than *τ*_1_, indicating that the binding of Dectin-1 to laminarin was substantially stabilized through positive cooperative interactions when neighboring Dectin-1 molecules were present on the same chain.

These observations provide direct real-time evidence for cooperative assembly of Dectin-1 on β-glucan chains. Although HS-AFM does not directly determine the stoichiometry of the resulting complexes, the observed cooperative stabilization is consistent with the 3:3 Dectin-1 assembly established independently by native MS. The MS data demonstrated that three Dectin-1 molecules are incorporated into each complex and that laminarin chains of DP 25–36 are preferentially enriched within the assembly. The HS-AFM results suggest that such multivalent engagement stabilizes the interaction, probably through cooperative binding along the glucan chain.

Together with our FIDA measurements indicating cooperative binding behavior (Hill coefficient >1), these HS-AFM data support a model in which initial monomeric binding of Dectin-1 to laminarin is transient, but subsequent recruitment of additional Dectin-1 molecules leads to the formation of a stable multimeric complex. This multivalent assembly may reduce Dectin-1 dissociation by distributing mechanical and thermal fluctuations across multiple receptor–ligand contacts.

While discrete trimeric assemblies were not directly resolved by HS-AFM, the technique clearly revealed cooperative recruitment and stabilization of Dectin-1 on laminarin chains, consistent with the multivalent assembly inferred from the native MS and FIDA analyses. However, because laminarin was immobilized on mica prior to Dectin-1 addition, the possibility remained that steric occlusion of part of the glucan surface might limit the accessibility of all receptor-binding sites.

### High-speed AFM observation of Dectin-1–scleroglucan complex formation

To determine whether the apparent 3:3 stoichiometry observed for laminarin simply reflected the limited length of this relatively short β-glucan, we examined the interaction of Dectin-1 with scleroglucan, a substantially longer β-(1→3)-glucan containing β-(1→6)-linked monoglucosyl side chains^28^.

**Fig. 9:**
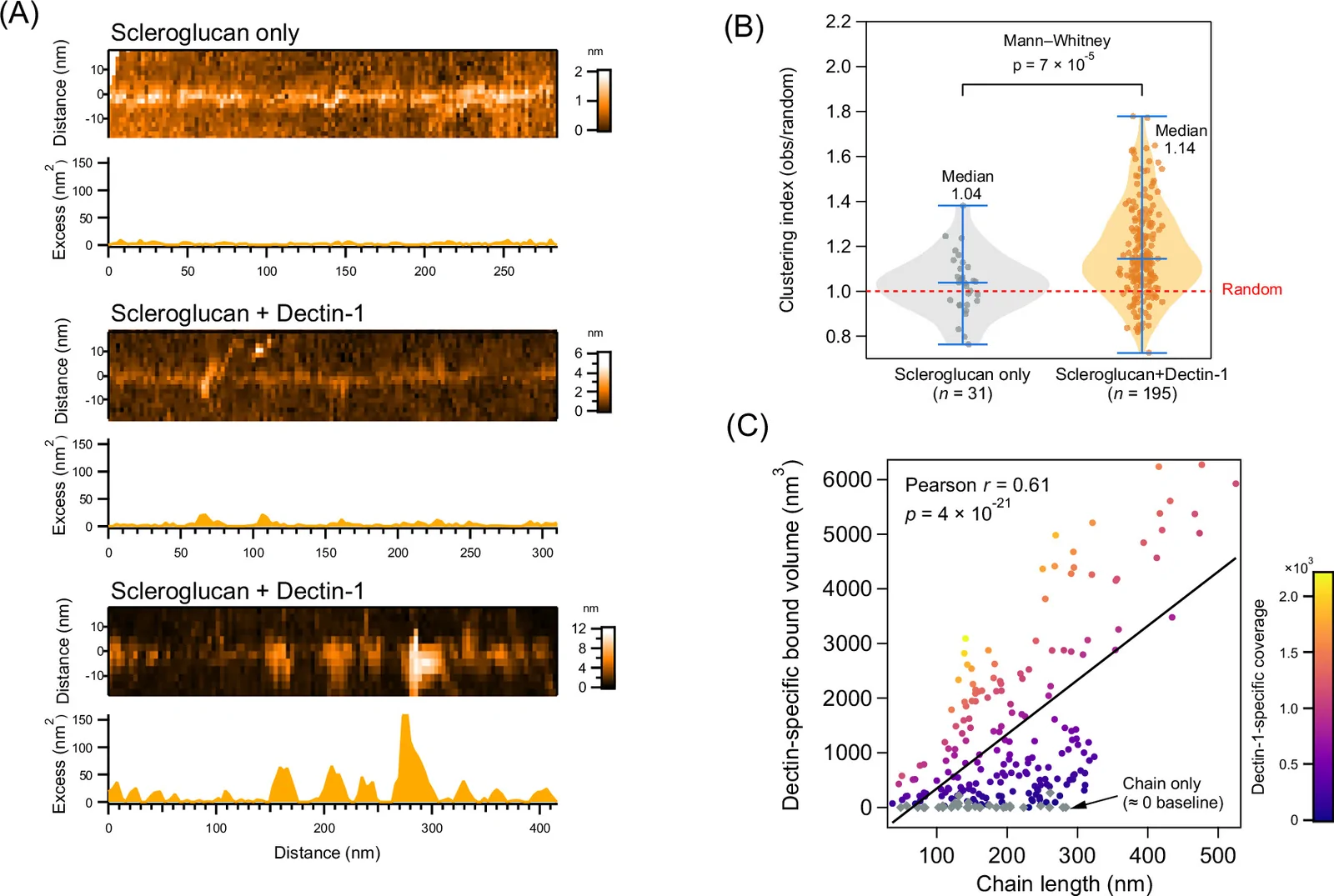
Dectin-1 undergoes cooperative clustering on individual scleroglucan chains as revealed by HS-AFM. **A** Representative chain-aligned box images and corresponding excess-volume profiles. Control scleroglucan chains exhibited nearly uniform profiles (top; cluster index = 1.04), whereas Dectin-1–bound chains displayed localized peaks and extended high-volume patches at low (middle) and high (bottom) receptor occupancies. All profiles are plotted on a common vertical scale (0–180 nm²), allowing direct visualization of Dectin-1–dependent volume accumulation along the glucan chain. **B** Comparison of cluster indices between control and Dectin-1–bound chains. Control chains remained close to the random expectation (median, 1.04), whereas Dectin-1 binding significantly increased clustering (median, 1.14; two-sided Mann–Whitney test, *p* = 7 × 10⁻⁵), indicating that the observed clustering arises from receptor binding rather than intrinsic chain heterogeneity. **C** Dependence of Dectin-1 binding on scleroglucan chain length. Background-corrected Dectin-1–specific excess volume increased with chain length (Pearson *r* = 0.61, *p* = 4 × 10⁻²¹), demonstrating that longer glucan chains accommodate larger amounts of bound Dectin-1 after correcting for the polysaccharide contribution.

Time-resolved HS-AFM analysis showed progressive accumulation of Dectin-1 on individual scleroglucan chains during the observation period (Movie S4; Fig. S4A). The chain-aligned excess-volume profiles further showed that the Dectin-1 molecules were not distributed uniformly along the glucan chains but instead formed localized regions of elevated occupancy, appearing as discrete peaks and extended high-volume patches (Fig. 9A; Movie S5). Consistent with these visual observations, Dectin-1–bound chains exhibited significantly higher clustering indices as compared with control chains lacking protein (respective median values, 1.14 versus 1.04; *p* = 7 × 10⁻⁵; Fig. 9B), indicating that receptor accumulation occurs in a spatially non-random manner. Furthermore, the clustering index increased with Dectin-1 coverage (Fig. S4B), suggesting that receptor accumulation preferentially occurs through local cooperative recruitment rather than through independent random binding events.

We also examined whether receptor binding depended on glucan chain length. Quantitative analysis revealed a significant positive correlation between background-corrected Dectin-1–specific excess volume and scleroglucan chain length (Pearson *r* = 0.61, *p* = 4 × 10⁻²¹; Fig. 9C), demonstrating that longer β-glucan chains accommodate progressively larger amounts of bound Dectin-1.

Together, these observations demonstrate that Dectin-1 undergoes cooperative clustering on extended β-glucan chains and indicate that receptor clustering is not restricted to short laminarin molecules. The ability of long scleroglucan chains to support progressively larger receptor clusters argues against the possibility that the 3:3 stoichiometry observed for laminarin arises solely from steric limitations imposed by glucan length. In contrast to laminarin, the extended scleroglucan chains would be expected to interact with the mica surface through only limited contact regions, leaving a substantial fraction of the glucan surface accessible to Dectin-1 in solution. The observation of cooperative clustering under these conditions suggests that the observed behavior of laminarin cannot be explained solely by surface-induced steric occlusion and is instead consistent with an intrinsic propensity of Dectin-1 to undergo cooperative assembly on β-glucan chains.

### Chemical crosslinking supports ligand-induced Dectin-1 oligomerization

The above HS-AFM analyses demonstrated cooperative clustering of Dectin-1 on β-glucan chains, but it remained unclear whether this cooperativity was accompanied by receptor–receptor association or mediated exclusively through ligand-dependent effects on the glucan scaffold. To differentiate between these possibilities, we performed chemical crosslinking experiments using glutaraldehyde.

**Fig. 10:**
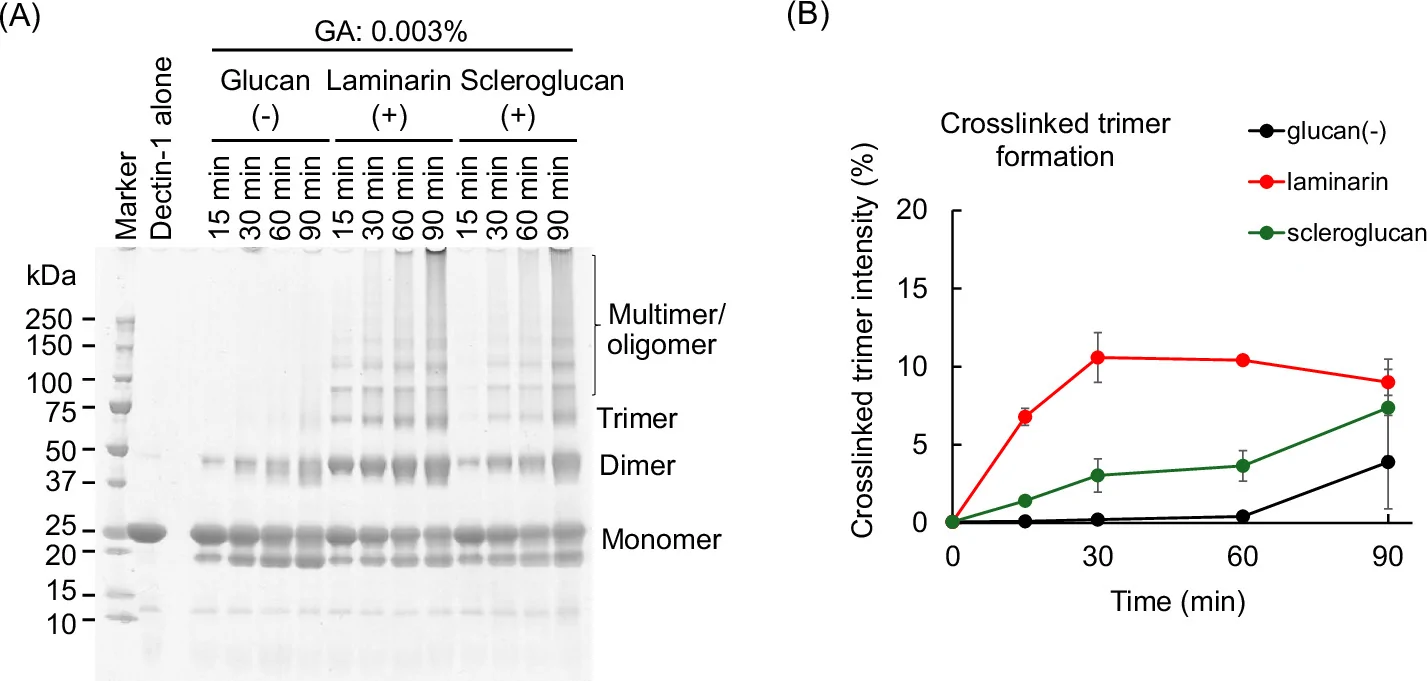
Ligand-dependent chemical crosslinking of Dectin-1 by glutaraldehyde. **A** Time-dependent glutaraldehyde (GA) crosslinking of Dectin-1 analyzed by SDS–PAGE in the absence or presence of laminarin or scleroglucan. The progressive appearance of higher-molecular-weight species indicates ligand-promoted oligomerization of Dectin-1. **B** Quantification of Dectin-1 trimer formation. Trimer abundance was determined from SDS–PAGE band intensities and is expressed as the fraction of the trimer band relative to the total signal from all detectable species. Data represent the mean trimer fraction ± S.D. from three independent experiments.

Purified Dectin-1 was incubated in the absence or presence of laminarin or scleroglucan, followed by time-dependent crosslinking and SDS–PAGE analysis (Fig. 10A). In the absence of β-glucan ligand, Dectin-1 remained predominantly monomeric throughout the reaction period, with only limited formation of oligomeric species; in contrast, both laminarin and scleroglucan promoted the appearance of oligomeric bands including dimeric and trimeric Dectin-1 species. Quantitative analysis of SDS–PAGE band intensities demonstrated that trimer formation was substantially enhanced by both glucans relative to the ligand-free control (Fig. 10B). Notably, laminarin consistently produced a higher fraction of trimeric Dectin-1 as compared with scleroglucan, suggesting that specific glucan structural features, including chain length and higher-order organization, influence the efficiency of receptor oligomerization. Whether this difference reflects glucan chain length, branching, or higher-order glucan organization remains to be determined.

These results provide independent biochemical evidence that β-glucan binding promotes the close spatial association of Dectin-1 molecules. While they do not exclude the possibility that glucan-mediated conformational effects contribute to cooperativity, the ligand-dependent formation of crosslinked Dectin-1 oligomers indicates that receptor–receptor proximity is an important component of the assembly process. Together with the native MS, FIDA, and HS-AFM analyses, the crosslinking data support a model in which β-glucan ligands drive cooperative receptor clustering and stabilize higher-order assemblies of Dectin-1. The enhanced formation of crosslinked trimers, especially in the presence of laminarin, is also consistent with the predominant 3:3 Dectin-1–laminarin assembly identified by native MS.

Nevertheless, several important issues remain unresolved. First, the β-glucans analyzed in this study were heterogeneous with respect to both chain length and branching pattern. The scleroglucan experiments demonstrated that extended glucan chains can support further cooperative recruitment of Dectin-1, but the individual contributions of chain length and β-(1→6) branching could not be differentiated. Second, all experiments were performed using soluble recombinant Dectin-1 ectodomains rather than the full-length transmembrane receptor. While this reductionist approach facilitated the direct characterization of receptor–glucan interactions and assembly behavior in solution, it does not fully reproduce the geometric constraints imposed by membrane anchoring. Consequently, the precise architecture of the assemblies observed here may differ from that formed in the native cellular context. Lastly, although the present study has established a 3:3 stoichiometry for the Dectin-1–laminarin complex, it does not provide sufficient constraints to derive a unique 3D structural model. The relative arrangement of Dectin-1 molecules and glucan chain(s) within the assembly therefore remains unresolved.

Future studies employing structurally defined β-glucans together with membrane-reconstituted, cell-based, biochemical, and structural approaches will be required to define the architecture of the assembly and determine how glucan structure and membrane confinement influence receptor assembly and signaling. Such studies should further clarify how lectin receptors decode structurally heterogeneous glycan ligands.

## Conclusion

**Fig. 11:**
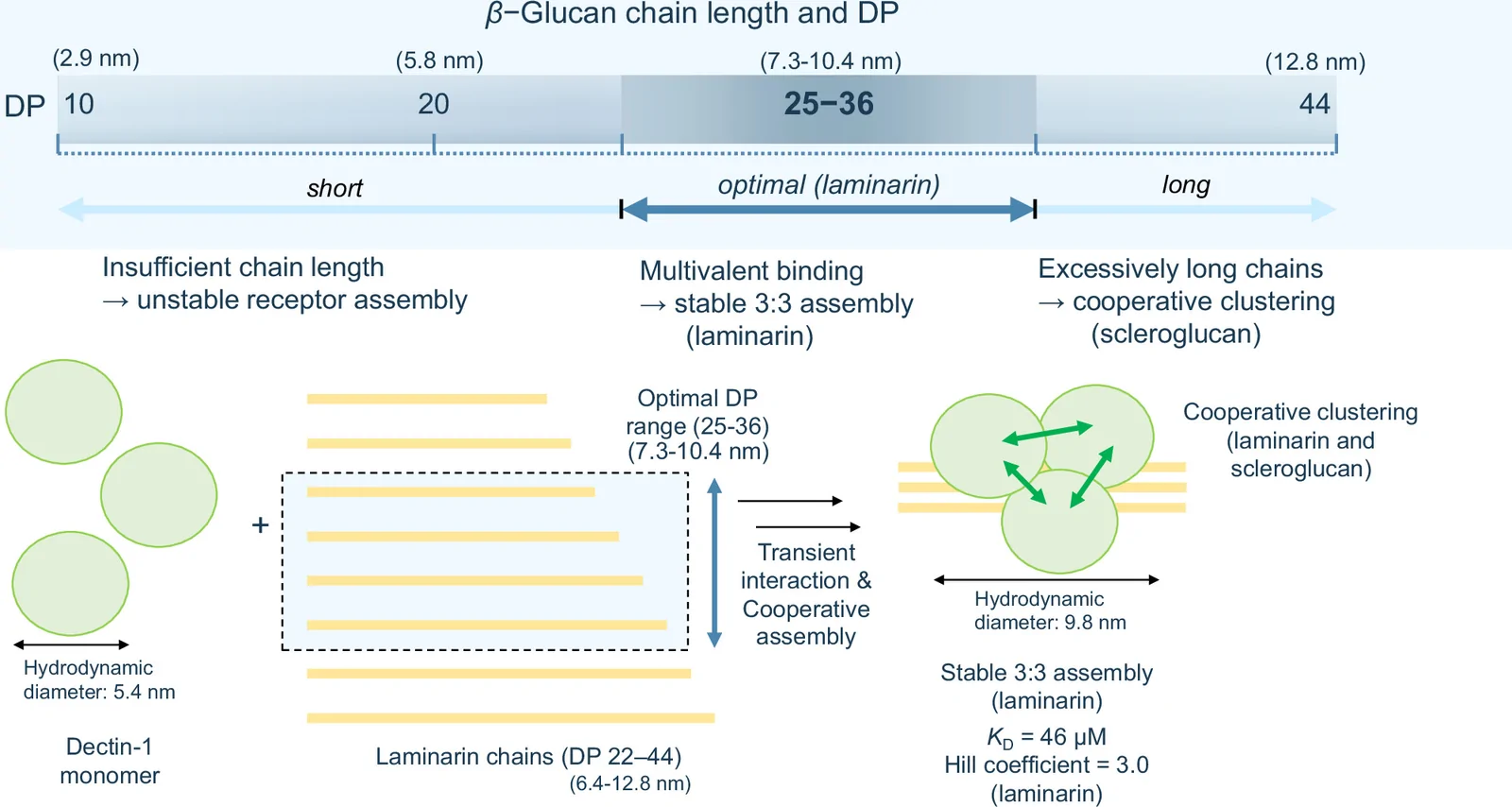
Proposed model of chain-length–dependent β-glucan recognition and cooperative Dectin-1 assembly. Monomeric Dectin-1 (hydrodynamic diameter, 5.4 nm) binds heterogeneous laminarin chains (DP 22–44) in a chain-length–dependent manner. Contour lengths were estimated by assuming a helical rise of 0.29 nm per glucose residue based on the X-ray diffraction parameters of β-1,3-glucan^16,24^. Chains within an optimal length window (DP 25–36; contour length, 7.3–10.4 nm) are selectively enriched in the stable 3:3 complex (hydrodynamic diameter, 9.8 nm; *K*_*D*_ = 46 μM; Hill coefficient = 3.0), whereas shorter chains fail to stabilize receptor assemblies, resulting in predominantly transient interactions. Longer β-glucan chains such as scleroglucan can support additional cooperative recruitment of Dectin-1. Together, these observations support a molecular ruler mechanism in which β-glucan chain length regulates receptor occupancy and cooperative assembly, facilitating selective recognition of structurally heterogeneous β-glucan populations.

The present study has explored the molecular basis of Dectin-1 recognition of β-glucans using a combination of structural approaches. Native MS established the preferred stoichiometry of the laminarin-bound complex, FIDA demonstrated the positive cooperativity of complex formation, HS-AFM directly showed cooperative receptor clustering, and chemical crosslinking provided independent evidence of ligand-induced receptor association. Together, these analyses converge on a consistent mechanistic picture of β-glucan recognition by Dectin-1 in which laminarin chains within a defined chain-length range are preferentially enriched in a 3:3 Dectin-1–laminarin assembly, while cooperative receptor recruitment and stabilization occur on both short and extended β-glucan chains (Fig. 11).

Our findings suggest that Dectin-1 preferentially forms stable receptor assemblies on β-glucans of a defined chain length. In this framework, structurally heterogeneous β-glucan populations are effectively filtered through chain-length–dependent receptor organization, providing a molecular ruler mechanism by which specific biological information can be extracted from heterogeneous glycan ligands. Although the precise architecture of the receptor assembly remains to be established, our results support a model in which β-glucan length is a key determinant of productive Dectin-1 clustering and complex stabilization.

Future studies will be required to determine the molecular architecture underlying the observed 3:3 Dectin-1–laminarin stoichiometry and to elucidate how glucan length, branching, and membrane confinement influence receptor organization and downstream signaling in the cellular context.

## Materials and methods

### Materials

Uniformly labeled ^13^C_6_-d-glucose (99% atom ^13^C) was obtained from Cambridge Isotope Laboratories (Tewksbury, MA, USA). Laminarin derived from *Laminaria digitata* was obtained from Sigma-Aldrich (St. Louis, MO, USA). Scleroglucan from *Sclerotium rolfsii* was obtained from InvivoGen (San Diego, CA, USA). TEV protease was obtained from Nippon Gene (Tokyo, Japan). Glutaraldehyde was purchased from Nacalai Tesque (Kyoto, Japan).

### Expression of unlabeled and ^13^C-labeled mouse Dectin-1 lectin domain

The soluble mouse Dectin-1 lectin domain fused to an N-terminal protein G B1 domain (hereafter referred to as Dectin-1) was expressed via a pCold-TEV vector in *Escherichia coli* BL21(DE3) as previously described^29,30^. Glycerol stocks were streaked onto LB agar plates containing 50 µg/mL of ampicillin and incubated at 37 °C for 12–16 h. A single colony was used to inoculate 100 mL of LB medium (1.0 g of tryptone, 0.5 g of yeast extract, 1.0 g of NaCl per 100 mL) supplemented with 50 µg/mL of ampicillin, and the culture was grown at 37 °C for 8 h with shaking (200 rpm).

For unlabeled protein expression, the pre-culture was transferred into 800 mL of LB medium with 50 µg/mL of ampicillin and grown at 37 °C until an OD_600_ of 1.0 (NanoDrop One, Thermo Fisher Scientific). The culture was cooled to 15 °C, induced with 0.1 mM IPTG, and incubated overnight at 15 °C with shaking (200 rpm).

For uniformly ^13^C-labeled protein expression, the pre-culture was harvested (2000 × *g*, 10 min), resuspended, and inoculated into 1.0 L of M9 minimal medium (per 250 mL: 4.0 g of Na₂HPO₄·12H₂O, 0.5 g of KH₂PO₄, 0.125 g of NaCl, 0.25 g of NH₄Cl), supplemented with 2 mM MgSO₄, 21.6 µM FeCl₂, 1.2 mg/L of biotin, 2.4 mg/L of thiamine, 100 µM CaCl₂, 2 g/L of uniformly labeled ^13^C_6_-glucose, and 50 µg/mL of ampicillin. Growth and induction were performed as described above.

Cultured bacterial cells (200 mL) were harvested by centrifugation (13,680 *g*, 4 °C, 10 min) and resuspended in 10 mL of BugBuster Protein Extraction Reagent (Millipore). After incubation at 25 °C with shaking (100 rpm, 20 min), 10 mL of PBS containing 0.1% (v/v) Triton X-100 was added, and cells were disrupted by ultrasonication (20 kHz; 1 s on/off cycles; 2 min × 3). The lysate was clarified by centrifugation, and the pellet was washed with 40 mL of PBS with 10% (v/v) BugBuster. The washed pellet was solubilized in urea buffer (8 M urea, 50 mM Tris-HCl [pH 8.0], 50 mM NaCl) and centrifuged (20,150 × *g*, 30 min), and the supernatant was collected for refolding.

The denatured protein solution was passed through a 0.45-µm filter and slowly added dropwise to refolding buffer (1.0 M Tris-HCl [pH 8.0], 0.4 M l-arginine, 5 mM reduced glutathione, 0.5 mM oxidized glutathione) at a ratio of 10 mL of protein solution per 1.0 L of refolding buffer. The mixture was stirred at 4 °C for 24–48 h to allow refolding.

Refolded protein was concentrated using an ultrafiltration system (Quixstand benchtop, GE Healthcare) equipped with a 10-kDa MWCO membrane (Kvick Lab, GE Healthcare) prewashed with 1.0 L of 0.2 M NaCl. The solution was centrifugally concentrated (2000 × *g*, 15 min, Suprema21, TOMY) and subjected to three sequential buffer exchanges with 500 mL of Tris buffer (20 mM Tris-HCl [pH 7.4], 150 mM NaCl, 1 mM CaCl₂). The pooled fractions were dialyzed against 2.0 L of the same buffer using a 3.5-kDa MWCO membrane (Spectrum Laboratories) with three buffer changes.

### Purification of recombinant proteins

Recombinant proteins were purified using Ni²⁺-charged Sepharose 6 Fast Flow resin (Cytiva) packed in a column pre-equilibrated with 20 mM Tris-HCl (pH 7.4), 150 mM NaCl, and 1 mM CaCl₂. The dialyzed protein solution was filtered through a 0.45-µm membrane and loaded on the column. The column was then washed with 15 mL of wash buffer (20 mM Tris-HCl [pH 7.4], 150 mM NaCl, 1 mM CaCl₂, 20 mM imidazole). Bound proteins were eluted stepwise in 6-mL fractions of elution buffer containing 100–500 mM imidazole in the same buffer. Fractions exhibiting a single ~25-kDa band by SDS–PAGE were pooled and dialyzed extensively against 100 volumes of Tris buffer using a 3.5-kDa MWCO dialysis membrane.

### TEV protease cleavage

The dialyzed protein sample was incubated with TEV protease (Nippon Gene, Tokyo, Japan) at a ~1:1 (w/w) ratio in the presence of 5 mM reduced glutathione and 0.5 mM oxidized glutathione at 4 °C for 48–72 h. Following cleavage, the reaction mixture was filtered through a 0.45-µm membrane and applied to a Ni²⁺-affinity column pre-equilibrated as described above. The flow-through fraction was collected and analyzed by SDS–PAGE followed by Coomassie Brilliant Blue staining.

### Calculations of the theoretical molecular weight of Dectin-1

The following amino acid sequence of TEV protease-cleaved Dectin-1 was used for molecular weight calculation:

GSTYKLILNGKTLKGETTTEAVDAATAEKVFKQYANDNGVDGEWTYDDATKTFTVTEGGFSQSCLPNWIMHGKSCYLFSFSGNSWYGSKRHCSQLGAHLLKIDNSKEFEFIESQTSSHRINAFWIGLSRNQSEGPWFWEDGSAFFPNSFQVRNTVPQESLLHNCVWIHGSEVYNQICNTSSYSICEKELEFKLVDLQSR.

The sequence contains six cysteine residues, which were assumed to form three intramolecular disulfide bonds under native conditions. The theoretical average molecular weight of oxidized Dectin-1 was calculated to be 22,554 Da, corresponding to the molecular formula C_1007_H_1491_N_267_O_312_S_7_ (C, 12.0107; H, 1.00794; N, 14.0067; O, 15.9994; S, 32.065), based on IUPAC standard atomic weights^31^.

### Native and acid-denatured MS

Laminarin, unlabeled Dectin-1, and uniformly ^13^C-labeled Dectin-1 were buffer-exchanged into 150 mM ammonium acetate (pH 6.8) using a Micro Bio-Spin^TM^ P-6 gel column (Bio-Rad) according to the manufacturer’s instructions. MS measurements were performed using a Q Exactive UHMR Hybrid Quadrupole–Orbitrap Mass Spectrometer (Thermo Fisher Scientific)^32,33^. Samples (~3 µL) were loaded into gold-coated glass capillaries made in-house and subjected to nano-electrospray ionization. For native MS of Dectin-1 and Dectin-1–laminarin complexes, spectra were acquired in positive ion mode with the following instrument parameters: capillary voltage, 1.20 kV; detector mode, low *m*/*z*; ion transfer target, low *m*/*z*; resolution, 3125–6250; injection time, 200 ms; and trap gas pressure setting, 5.0. For laminarin, the trap gas pressure setting was 2.0, and the capillary voltage, only in negative ion mode, was changed to −1.00 kV. External mass calibration was performed using 2 mg/mL of cesium iodide in a solution of 50% 2-propanol and 50% water (v/v).

For acid-denatured MS, the buffer-exchanged samples used for native MS were treated with 30% (v/v) formic acid prior to analysis to disrupt noncovalent interactions. Denaturing spectra were acquired under identical instrumental settings except for a trap gas pressure setting of 2.0. Raw spectra were processed and deconvoluted using UniDec^34^.

### Flow-induced dispersion analysis

FIDA^35,36^ was performed to determine the dissociation constant, stoichiometry, and cooperativity of the Dectin-1–laminarin interaction under solution conditions. Measurements were conducted using a Fida1 instrument (Fida Biosystems).

Separations were carried out in a standard fused silica capillary (internal diameter, 75 μm, total length, 100 cm; Fida Biosystems), which was dynamically coated before use with a Dynamic HS coating reagent (Fida Biosystems) to minimize surface adsorption. The capillary chamber was maintained at 25 °C throughout the measurements. Samples containing protein and ligand were stored in the autosampler tray at 5 °C (Tray 1), while buffer solutions were maintained at 25 °C (Tray 2).

Purified Dectin-1 was used as the analyte at a final concentration of 3 μM in assay buffer (20 mM Tris-HCl [pH 7.4], 150 mM NaCl, 1 mM CaCl_2_). Laminarin was prepared as a series of dilutions (0.58, 1.17, 2.34, 4.68, 9.375, 18.75, 37.5, 75, and 150 μM) in the same buffer. For each measurement, Dectin-1 and laminarin were pre-mixed at the indicated concentrations and incubated at 25 °C to allow equilibration prior to analysis. The pre-mixed samples were applied as the indicator solution.

Detection was performed using an LED-based fluorescence detector (280 nm module, excitation at 275 nm; Fida Biosystems), which monitored intrinsic protein fluorescence. In FIDA, a narrow plug of the indicator solution is injected into a laminar flow within the capillary, and molecular diffusion leads to temporal broadening of the sample zone. The injection conditions for FIDA are summarized in Table 1.

**Table 1:** Method sequence for FIDA measurements

Inlet tray	Inlet vial	Pressure [mbar]	Time [s]	Measure	Comment
Tray 2	2	3500	20	No	Assay buffer + equilibration
Tray 1	Analyte	3500	30	No	Fill with analyte sample
Tray 1	Indicator	50	10	No	Inject indicator
Tray 1	Analyte	400	180	Yes	Mobilize and measure

The apparent diffusion coefficient (*D*) was determined based on Taylor–Aris dispersion theory^37,38^, where the temporal variance (${\sigma }^{2}$) of the elution profile is related to the mean residence time (${t}_{R}$) and the capillary radius (a=37.5 μm) according to

$$D=\frac{{a}^{2}}{24{\sigma }^{2}}{t}_{R}.$$

The hydrodynamic radius (${R}_{h}$) was subsequently calculated using the Stokes-Einstein equation:

$${R}_{h}=\frac{{k}_{B}T}{6\pi \eta D},$$

where ${k}_{B}$ is the Boltzmann constant, T is the absolute temperature (298 K, corresponding to the capillary temperature of 25 °C), and η is the solvent viscosity. Thus, complex formation between Dectin-1 and laminarin results in a decrease in the diffusion coefficient and a corresponding increase in the hydrodynamic radius.

Binding curves were constructed by plotting ${R}_{h}$ as a function of laminarin concentration. The data were fitted using non-linear regression based on the Hill equation^22,23^:

$${R}_{h}={R}_{h,{free}}+\frac{({R}_{h,{bound}}-{R}_{h,{free}}){[L]}^{n}}{{K}_{D}^{n}+{[L]}^{n}}\,,$$

where ${R}_{h,{free}}$ and ${R}_{h,{bound}}$ represent the hydrodynamic radii of free and fully bound Dectin-1, respectively, ${K}_{D}$ is the apparent dissociation constant, n is the Hill coefficient, and [L] is the laminarin concentration. Curve fitting was performed using GraphPad Prism 7 (Dotmatics). All measurements were performed in triplicate (*n* = 3).

Stoichiometry inferred from FIDA was compared with values obtained from native MS. Theoretical hydrodynamic radii were also estimated from the crystal structure of the murine Dectin-1 lectin domain (PDB ID: 2BPD)^14^ using the Structural PDB Rh predictor (Fida Biosystems) after removal of bound water molecules.

### High-speed atomic force microscopy

HS-AFM was performed as in previous reports^39,40^. All measurements were carried out in PBS (pH 7.4) at room temperature. To characterize individual components, Dectin-1 alone (1 μM) and laminarin alone (100 μM) were each deposited onto freshly cleaved mica and observed by HS-AFM. For observation of Dectin-1–laminarin interactions, laminarin (100 μM) was first adsorbed onto mica, after which Dectin-1 was introduced into the liquid cell at a final concentration of 0.3 μM. For observation of Dectin-1–scleroglucan interactions, scleroglucan (2.58 mg/mL) was adsorbed onto mica, and then Dectin-1 was added at a final concentration of 0.7 μM. Time-resolved HS-AFM imaging was performed in tapping mode using small cantilevers optimized for high-speed scanning. Image analysis was performed using laboratory-built AFM analysis software (pyNuD) to evaluate changes in complex formation and dissociation over time.

### Chemical crosslinking with glutaraldehyde

Chemical crosslinking experiments were performed to assess ligand-induced oligomerization of Dectin-1. Purified mouse Dectin-1 was diluted in PBS (pH 7.4) to a final concentration of 0.75 mg/mL. Laminarin was added at a 10-fold molar excess (final concentration, 1.3 mg/mL). Scleroglucan was added at the same mass concentration as laminarin (1.3 mg/mL) to achieve a comparable concentration of glucose residues in the two β-glucan preparations. Glutaraldehyde was diluted in PBS and added to the protein solution at a final concentration of 0.003% (v/v). Crosslinking reactions were carried out in a reaction volume of 20 μL at room temperature. After 15, 30, 60, and 90 min of incubation, 5 μL of the reaction mixture was withdrawn and immediately quenched by addition of 1 μL of 1 M Tris-HCl (pH 7.4) to give a final concentration of 0.17 M. The samples were analyzed by SDS–PAGE, and band intensities corresponding to monomeric and oligomeric species were quantified using Image Lab software (Bio-Rad). The fraction of trimer was calculated as the integrated intensity of the trimer band divided by the summed intensities of all detectable Dectin-1 bands. Three independent experiments were performed; data are presented as mean ± S.D.

## Supplementary information

Transparent Peer Review file

Supplementary Information

Description of Additional Supplementary Files

Movie S1

Movie S2

Movie S3

Movie S4

Movie S5

Supplementary Data 1

Supplementary Data 2

Supplementary Data 3

## Data Availability

The source data for Figs. 6, 7 and 10 are provided in Supplementary Data 1–3, respectively, accompanying this article. All other data supporting the findings of this study are available from the corresponding author upon reasonable request.
