# Supplementary material for: Chain-length-selective cooperative assembly underlies β-glucan recognition by Dectin-1
Source: Commun Chem. 2026 Nov 10;9:332. doi: 10.1038/s42004-026-02217-x (PMC13751592; doi:10.1038/s42004-026-02217-x)
Supplement: Supplementary file 1 [file 42004_2026_2217_MOESM1_ESM.pdf]

nature portfolio

Peer Review File

Chain-length–selective cooperative assembly underlies β-glucan recognition by Dectin-1

Corresponding Author: Professor Yoshiki Yamaguchi

This file contains all reviewer reports in order by version, followed by all author rebuttals in order by version.

Version 0:

Reviewer comments:

Reviewer #1

(Remarks to the Author)
Manabe, et al addresses the important biophysical question of how Dectin-1 interacts with beta-1,3-glucan, forming macromolecular assemblies whose fine structure may impact cell signaling and functional response downstream of Dectin-1. The work utilizes relevant methods (MS, FIDA, AFM), all competently performed. The paper is also very lucid and logically arranged. In my opinion, it does represent a helpful contribution to the literature in this field.
The use of the term "trimer" to describe Dectin-1's interaction with beta-glucan is moderately problematic. This term's typical use connotes an ordered molecular assembly of three entities (i.e., Dectin-1), not merely a stoichiometry involving three copies. I am not convinced an ordered trimer is unambiguously proven by the data presented. The existence of cooperativity (FIDA) and appearance of a long half-life for Dectin-1 neighbors (AFM) might suggest an ordered protein trimer, but isn't it possible that the cooperativity could work through a mechanism as follows: Dectin-1 binding induces a glucan conformational shift that improves binding of subsequent Dectin-1 copies to the same glucan (without the Dectin-1s necessarily being in an ordered protein-protein assembly)? Also, if the minimal glucan DP required to support Dectin-1 binding is ~10 and the DP of glucans used in the study is ~30, it is possible that apparent "trimers" of Dectin-1 merely reflect the maximum number of Dectin-1 molecules that can possibly bind to this small glucan. Perhaps larger DP glucans would simply show a proportionally larger number of Dectin-1 bound (i.e., as "beads on a string") rather than increasing Dectin-1 binding in multiples of three (as would be expected if a trimer was the basic unit of Dectin-1/glucan interaction). I think the authors should be cautious about the use of the term "trimer" or at least make it clear that whether this is an ordered macromolecular trimeric assembly of Dectin-1 or only an empirically observed stoichiometry is not yet fully established.
This work was accomplished with soluble Dectin-1 fusion proteins, but of course, Dectin-1 is normally expressed and functions as a transmembrane protein. Therefore, it is possible that there are bound Dectin-1/glucan configurations that are accessible to soluble Dectin-1 that would be sterically inaccessible if the protein were present in its transmembrane state. This is a limitation of the methods used, which I think are nevertheless helpful to the field. In any case, I believe the authors should more clearly state the caveat that it cannot be established, based on this work, that the "trimeric" interaction observed does actually exist for Dectin-1 in its native cell membrane context.
One might have expected to see "trimers" of Dectin-1 on mica-bound glucans in AFM. I do not believe there are any clear examples of this in the data presented. Do the authors have an explanation of why triplets of Dectin-1 on glucans are not evident in their AFM data? Is this possibly a steric effect of binding the glucan to a surface, then attaching Dectin-1? Perhaps the third binding site is on the inaccessible surface apposed side of the glucan. If glucan/Dectin-1 complexes were formed in solution and the material adsorbed to mica for AFM, would you then see glucans with three Dectin-1 copies bound?
I appreciate the opportunity to review the authors' interesting report and hope the above contributions will be helpful in further consideration of the work.

Reviewer #2

(Remarks to the Author)
The manuscript analyzes the interaction between Dectin-1, a monomeric C-type lectin, and laminarin, a polysaccharide consisting of linear b-glucans with presence of branches. The authors demonstrated previously the cooperative

oligomerization of Dectin-1 on this substrate and they now confirm their results using different biophysical approaches, such as isotopic mass spectrometry, flow-induced dispersion analysis and high-speed atomic force microscopy. The manuscript reads well and is technically sound, but the results represent only a marginal increment in our knowledge of the interaction. In the absence of variation on the length and nature of the polysaccharide substrate and without proposal of a model of interaction, the results do not represent a sufficient advance for publication.

The isotopic mass spectrometry is of interest and the use of 13C-labelled protein elegantly establishes the stoichiometry for the selected substrate. The description of result is lengthy with too much details for the already established knowledge (presence of calcium).

The flow-induced dispersion analysis confirms the oligomerization but with no details on stoichiometry and the information from the Hill plot are redundant with those published by the same group. Mass photometry could be considered for getting information on the MW of the complexes.

The AFM movies are of interest, but again only confirms that dectin-1 has a tendency to cooperatively binds to laminarin. The images do not confirm the stochiometry.

The weak point of the manuscript is the use of commercial substrate without control of size or degree of branching. Variation in the composition of this polysaccharide (branding degree) could explain differences with previously obtained data. Use of longer polysaccharides would demonstrate the length needed for getting additional Dectin-1 on the surface. Use of linear b-glucan would demonstrate the effect of branching.

Finally, the article would be much stronger with some attempts to propose a 3D model of the complex. The authors state that a helix model of laminarin has been used for rationalizing the AFM images, but the model is not shown or validated. The structure of laminarin is still controversial (single or triple helix) and this is not discussed.

Reviewer #3

(Remarks to the Author)
This manuscript focuses on the characterization of the interaction between the Dectin-1, a lectin involved in innate immunity, and laminarin, a beta-glucan polysaccharide, being beta-glucans the known natural ligands of this lectin. The basis for this interaction are currently not fully understood.
The authors use three different techniques to obtain different types of information. The application of these techniques to this system for the first time (to the best of my knowledge) provides new data on this complex interaction, allowing the authors to propose a novel interaction model. The most significant findings is that the binding imply complexes of stoichiometry 3:3, that there is a preferred laminarin length for the binding, and the existence of positive cooperativity. In my opinion these conclusions are novel, and they will be of interest to the community.
A couple of issues are not clear:
- In the MS analysis of laminarin: in Figure 3A it is said that deconvolution of the positive ion mode spectrum was not possible due to overlapping charge sates), and it is actually not shown (contrary to the negative ion mode spectrum), while in the manuscript (page 5, lines 144-147) it is stated that “the positive-ion spectrum showed a similar distribution, with a DP of 22-24” where this analysis come from if deconvolution is not possible?
- In the high-speed AFM, the interpretation of the authors is that laminarin form a helix, based on a simulated AFM from a model structure of a laminarin helix. How was this structural model generated? Please provide more data: is this a single helix? Laminarin has been proposed to oligomerize, is this taken into account here?

Version 1:

Reviewer comments:

Reviewer #1

(Remarks to the Author)
I am satisfied with the responses and revisions the authors made to critiques, and I am in support of publication at this time.

Reviewer #2

(Remarks to the Author)
The authors provided extensive and well argumented answers to the reviewer questions. The performed new experiments and modified the manuscript has requested.

Reviewer #3

(Remarks to the Author)
The authors have satisfactorily addressed my two concerns. The MS data interpretation has been further clarified (this is also now included in the manuscript). Additionally, simulated AFM images for single-chain glucans have been included, which rule out their major presence, and reinforce the proposed multi-chain helical assembly, while at the same time

moderating the interpretation of the Dectin-1 recognised glucan assembly.

Open Access This Peer Review File is licensed under a Creative Commons Attribution 4.0 International License, which permits use, sharing, adaptation, distribution and reproduction in any medium or format, as long as you give appropriate credit to the original author(s) and the source, provide a link to the Creative Commons license, and indicate if changes were made.
In cases where reviewers are anonymous, credit should be given to 'Anonymous Referee' and the source.
The images or other third party material in this Peer Review File are included in the article's Creative Commons license, unless indicated otherwise in a credit line to the material. If material is not included in the article's Creative Commons license and your intended use is not permitted by statutory regulation or exceeds the permitted use, you will need to obtain permission directly from the copyright holder.
To view a copy of this license, visit https://creativecommons.org/licenses/by/4.0/

Response letter

Reviewer #1 (Remarks to the Author):

1) Manabe, et al addresses the important biophysical question of how Dectin-1 interacts with beta-1,3-glucan, forming macromolecular assemblies whose fine structure may impact cell signaling and functional response downstream of Dectin-1. The work utilizes relevant methods (MS, FIDA, AFM), all competently performed. The paper is also very lucid and logically arranged. In my opinion, it does represent a helpful contribution to the literature in this field.

We thank the reviewer for their positive evaluation of our study and for recognizing its potential contribution to understanding the molecular basis of Dectin-1–β-glucan recognition and assembly.

2) The use of the term “trimer” to describe Dectin-1’s interaction with beta-glucan is moderately problematic. This term’s typical use connotes an ordered molecular assembly of three entities (i.e., Dectin-1), not merely a stoichiometry involving three copies. I am not convinced an ordered trimer is unambiguously proven by the data presented. The existence of cooperativity (FIDA) and appearance of a long half-life for Dectin-1 neighbors (AFM) might suggest an ordered protein trimer, but isn’t it possible that the cooperativity could work through a mechanism as follows: Dectin-1 binding induces a glucan conformational shift that improves binding of subsequent Dectin-1 copies to the same glucan (without the Dectin-1s necessarily being in an ordered protein-protein assembly)? Also, if the minimal glucan DP required to support Dectin-1 binding is ~10 and the DP of glucans used in the study is ~30, it is possible that apparent “trimers” of Dectin-1 merely reflect the maximum number of Dectin-1 molecules that can possibly bind to this small glucan. Perhaps larger DP glucans would simply show a proportionally larger number of Dectin-1 bound (i.e., as “beads on a string”) rather than increasing Dectin-1 binding in multiples of three (as would be expected if a trimer was the basic unit of Dectin-1/glucan interaction). I think the authors should be cautious about the use of the term “trimer” or at least make it clear that whether this is an ordered macromolecular trimeric assembly of Dectin-1 or only an empirically observed stoichiometry is not yet fully established.

We thank the reviewer for this insightful comment. In response, we have revised our manuscript to describe our finding as an experimentally observed 3:3 stoichiometry and to avoid claiming a definitive ordered trimeric architecture, instead clarifying that structural determination remains a future goal. In the revised manuscript, we have distinguished more clearly between experimentally established stoichiometry and inferred assembly organization, emphasizing throughout that the existence of an ordered trimeric arrangement remains a

working model rather than a definitive structural conclusion.

We have also performed new glutaraldehyde crosslinking experiments in the presence of both laminarin and scleroglucan (Page 12, Lines 390-408). These experiments revealed ligand-dependent formation of higher-order Dectin-1 oligomers, providing independent biochemical evidence that β-glucan binding promotes close spatial association of Dectin-1 molecules. While these data do not define the exact architecture or stoichiometry of the assembly, they support the interpretation that receptor–receptor interactions contribute to assembly formation. We have therefore explicitly discussed how receptor–receptor proximity contributes to the assembly process, but stated that glucan-mediated conformational effects cannot be excluded (Pages 12-13, Lines 409–413).

Lastly, to address the possibility that the apparent 3:3 stoichiometry simply reflects the limited size of laminarin chains, we performed new HS-AFM analyses using the substantially longer β-glucan scleroglucan (Pages 11-12, Lines 355-388). These experiments demonstrated progressive receptor accumulation and cooperative clustering along extended glucan chains, indicating that our observed recruitment of Dectin-1 to laminarin was not restricted to a maximum of three molecules due to glucan length; instead, our findings are compatible with continued receptor recruitment on extended glucan scaffolds. Thus, the observed 3:3 stoichiometry is unlikely to arise solely from steric constraints associated with short laminarin molecules.

3) This work was accomplished with soluble Dectin-1 fusion proteins, but of course, Dectin-1 is normally expressed and functions as a transmembrane protein. Therefore, it is possible that there are bound Dectin-1/glucan configurations that are accessible to soluble Dectin-1 that would be sterically inaccessible if the protein were present in its transmembrane state. This is a limitation of the methods used, which I think are nevertheless helpful to the field. In any case, I believe the authors should more clearly state the caveat that it cannot be established, based on this work, that the "trimeric" interaction observed does actually exist for Dectin-1 in its native cell membrane context.

We thank the reviewer for this important comment. We acknowledge that the present study was performed using soluble recombinant Dectin-1 ectodomains and therefore does not fully recapitulate the spatial constraints imposed by the native transmembrane receptor within the plasma membrane. Consequently, we cannot directly establish that the receptor assemblies that we observed would adopt identical architectures in the cellular membrane context.

To clarify this point, we have discussed this limitation in the revised manuscript (Page 13, Lines 424-429) and emphasized that our conclusions are restricted to the molecular recognition and assembly properties of the extracellular domain of Dectin-1 in solution.

Nevertheless, because β-glucan–induced Dectin-1 clustering has been reported previously in cellular systems, we believe that the cooperative assembly behavior observed here provides a mechanistic framework for understanding how β-glucans may promote receptor organization in vivo. Further studies using membrane-reconstituted or cell-based systems will be required to determine how membrane confinement influences the architecture and signaling properties of Dectin-1 assemblies.

4) One might have expected to see "trimers" of Dectin-1 on mica-bound glucans in AFM. I do not believe there are any clear examples of this in the data presented. Do the authors have an explanation of why triplets of Dectin-1 on glucans are not evident in their AFM data? Is this possibly a steric effect of binding the glucan to a surface, then attaching Dectin-1? Perhaps the third binding site is on the inaccessible surface apposed side of the glucan. If glucan/Dectin-1 complexes were formed in solution and the material adsorbed to mica for AFM, would you then see glucans with three Dectin-1 copies bound?
We thank the reviewer for this insightful comment. We agree that discrete trimeric assemblies are not directly resolved in the HS-AFM images. In the present study, HS-AFM was primarily used to visualize the dynamic behavior of Dectin-1 on β-glucan chains and to assess cooperative receptor recruitment rather than to determine the exact stoichiometry of individual complexes. We also agree that adsorption of laminarin onto the mica surface might potentially restrict access to some binding sites. As suggested by the reviewer, a fraction of the glucan surface may be oriented toward the mica substrate and thus be inaccessible to Dectin-1.

To address the reviewer's concerns, we have discussed these limitations in the revised manuscript (Page 11, Lines 348-353). In addition, we have performed additional HS-AFM analyses using scleroglucan, a substantially longer β-glucan that exhibits weaker surface confinement and greater conformational freedom on mica. These experiments revealed progressive receptor accumulation and cooperative clustering along extended glucan chains, indicating that receptor clustering is not restricted to the geometry of mica-adsorbed laminarin. Furthermore, we performed new glutaraldehyde crosslinking experiments, which independently demonstrated ligand-induced formation of Dectin-1 oligomers in solution (Pages 12-13, Lines 390-418).

We agree that direct visualization of preassembled Dectin-1–β-glucan complexes would be valuable. In our hands, however, Dectin-1 alone exhibited very weak adsorption to mica, making reliable observation of preformed complexes technically challenging. We therefore regard this as an important subject for future investigation.

5) I appreciate the opportunity to review the authors' interesting report and hope the above contributions will be helpful in further consideration of the work.

We thank the reviewer for their constructive comments and careful evaluation of our work. The suggestions have been very helpful in improving the manuscript. Although important questions regarding Dectin-1 assembly remain to be addressed, we believe that our findings increase our understanding of the molecular basis of Dectin-1–β-glucan recognition and assembly, and provide a strong foundation for future studies.

Reviewer #2 (Remarks to the Author):

1) The manuscript analyzes the interaction between Dectin-1, a monomeric C-type lectin, and laminarin, a polysaccharide consisting of linear b-glucans with presence of branches. The authors demonstrated previously the cooperative oligomerization of Dectin-1 on this substrate and they now confirm their results using different biophysical approaches, such as isotopic mass spectrometry, flow-induced dispersion analysis and high-speed atomic force microscopy. The manuscript reads well and is technically sound, but the results represent only a marginal increment in our knowledge of the interaction. In the absence of variation on the length and nature of the polysaccharide substrate and without proposal of a model of interaction, the results do not represent a sufficient advance for publication.

We thank the reviewer for this constructive feedback. Although we consider that the original findings represent a significant mechanistic advance, we acknowledge that the manuscript would be strengthened by additional evidence regarding the effects of glucan architecture and by a more explicit mechanistic model. In response, therefore, we have substantially revised the manuscript by (i) incorporating new HS-AFM analyses using the longer β-glucan scleroglucan, (ii) performing chemical crosslinking experiments that provide independent evidence for ligand-induced Dectin-1 oligomerization, and (iii) expanding the structural interpretation and mechanistic model of Dectin-1–β-glucan assembly (Fig. 11, Pages 23-24, Lines 788-800). We believe that these additions have significantly strengthened the mechanistic framework and broadened the impact of the study beyond our previous observations of cooperative binding.

2) The isotopic mass spectrometry is of interest and the use of 13C-labelled protein elegantly establishes the stoichiometry for the selected substrate. The description of result is lengthy with too much detail for the already established knowledge (presence of calcium).

We thank the reviewer for this suggestion. We agree that the Ca^{2+} dependence of Dectin-1 binding has been established previously and that our original description was unnecessarily detailed. Accordingly, we have shortened this section and limited the discussion to the

observations directly relevant to the present study (page 4, Lines 115-116).

3) The flow-induced dispersion analysis confirms the oligomerization but with no details on stoichiometry and the information from the Hill plot are redundant with those published by the same group. Mass photometry could be considered for getting information on the MW of the complexes.

We thank the reviewer for this helpful suggestion. We agree that FIDA does not directly provide stoichiometric information and that the primary evidence for the 3:3 Dectin-1–laminarin stoichiometry is obtained from native MS. In the revised manuscript, we have clarified that FIDA is used primarily to quantify cooperative assembly in solution and to provide an independent measurement of ligand-dependent complex formation.

In response to the reviewer's suggestion, we performed additional mass photometry experiments to assess the molecular weight of the Dectin-1–laminarin complexes. Under our experimental conditions, however, the expected complex mass (~80–85 kDa) was too close to the practical lower detection limit of the technique, and we were unable to resolve a distinct population corresponding to the proposed trimeric complex with sufficient confidence for quantitative analysis (**Figure R1**). Because the data do not permit an unambiguous interpretation, we have chosen not to include them in the revised manuscript.

Although the Hill coefficient obtained by FIDA is broadly consistent with our previous observations of cooperative binding, the present measurements serve a different purpose. In combination with the native MS determination of stoichiometry and the HS-AFM visualization of receptor assembly dynamics, the FIDA analysis provides independent solution-phase validation that the Dectin-1–β-glucan interaction is highly cooperative under the same experimental conditions used throughout this study.

Based on the reviewer’s comments, we have revised the manuscript to better distinguish and discuss the specific contributions and limitations of each experimental approach.

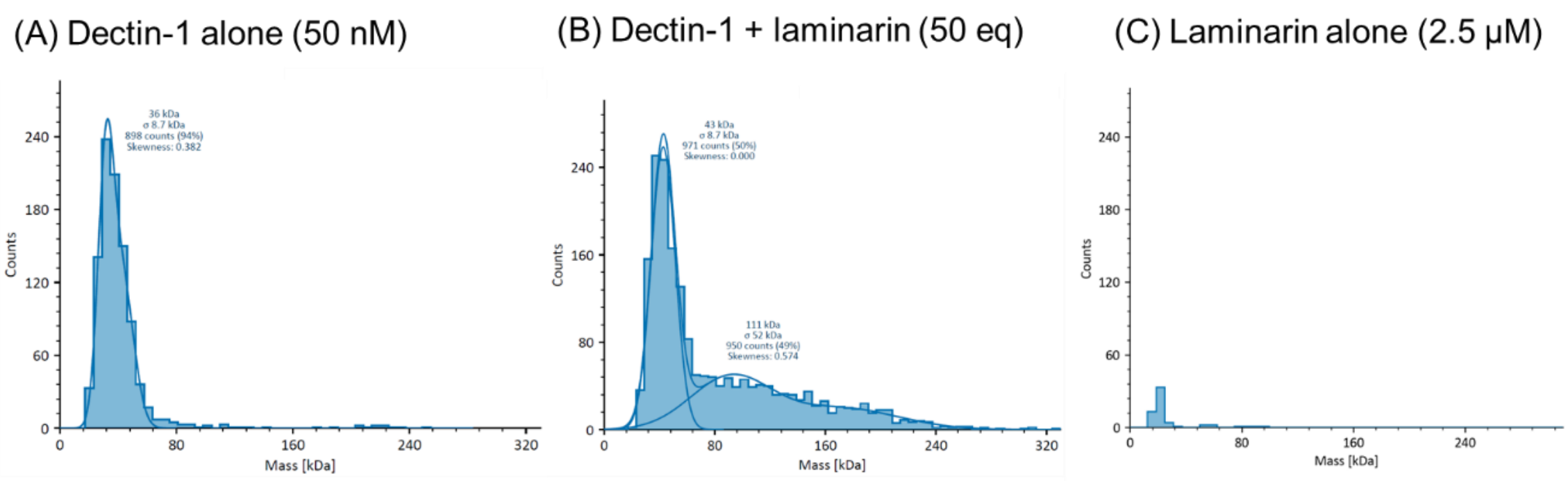

Figure R1 Mass photometry analysis of Dectin-1 in the absence and presence of laminarin.

(A) Mass photometry measurement of Dectin-1 alone (50 nM). A major population corresponding to monomeric Dectin-1 was observed. (B) Mass photometry measurement of Dectin-1 (50 nM) in the presence of laminarin (50-fold molar excess). (C) Mass photometry measurement of Laminarin alone (2.5 μM) corresponding to the same concentration in (B). In (B), in addition to the monomeric population, a broad higher-mass population consistent with the formation of Dectin-1–laminarin complexes was detected. However, the observed distribution was poorly resolved and located near the lower practical detection limit of the mass photometry method. Consequently, the data do not allow reliable determination of the molecular weight or stoichiometry of the complexes. These measurements therefore provide qualitative support for complex formation, but are not sufficiently quantitative for stoichiometric analysis.

4) The AFM movies are of interest, but again only confirms that dectin-1 has a tendency to cooperatively binds to laminarin. The images do not confirm the stoichiometry.

We thank the reviewer for this important comment. We agree that HS-AFM alone cannot determine the stoichiometry of the Dectin-1–β-glucan complex. Our intention was not to use AFM as evidence for the 3:3 stoichiometry itself, but rather to visualize the dynamic behavior and cooperative stabilization of receptor assemblies on individual β-glucan chains.

To further probe receptor oligomerization, we have performed additional chemical crosslinking experiments using glutaraldehyde and included the results as a new Figure 10 (pages 12-13, Lines 390-418). In the presence of both laminarin and scleroglucan, Dectin-1 exhibited enhanced formation of oligomeric species, including an enrichment of species consistent with crosslinked trimers; in contrast, only limited oligomerization was observed in the absence of ligand. These data provide independent biochemical evidence that β-glucan binding promotes close spatial association of Dectin-1 molecules.

To address the reviewer's concern regarding AFM, we have revised the manuscript to clarify that the 3:3 stoichiometry is established primarily by native MS, while HS-AFM provides complementary evidence for cooperative receptor clustering and stabilization. We have now explicitly stated that AFM does not directly determine stoichiometry, but reveals the dynamic assembly process that accompanies β-glucan recognition (Page 10, Lines 334-337).

5) The weak point of the manuscript is the use of commercial substrate without control of size or degree of branching. Variation in the composition of this polysaccharide (branching degree) could explain differences with previously obtained data. Use of longer polysaccharides would demonstrate the length needed for getting additional Dectin-1 on the

surface. Use of linear b-glucan would demonstrate the effect of branching.

We thank the reviewer for this important comment. We agree that both glucan chain length and branching frequency are potentially important determinants of Dectin-1 recognition and may contribute to differences among previous studies.

The objective of our present work was not to define the contribution of a single structural parameter using chemically homogeneous glucans, but rather to investigate how Dectin-1 recognizes ligands among naturally heterogeneous β-glucan populations. Laminarin was therefore intentionally selected because it represents a physiologically relevant soluble β-(1→3)-glucan preparation containing intrinsic heterogeneity in chain length and branching. This heterogeneity enabled us to directly examine whether Dectin-1 preferentially selects specific glucan species from a mixed population.

To address the reviewer's concern regarding chain-length limitation, we have performed additional HS-AFM and chemical crosslinking experiments using scleroglucan, a substantially longer β-(1→3)-glucan containing β-(1→6)-linked side chains. The new results show that Dectin-1 continues to accumulate cooperatively on extended glucan chains and that receptor occupancy increases with chain length (new Fig. 9 and Fig. S4). They demonstrate that the apparent 3:3 stoichiometry observed for laminarin is unlikely to arise solely from steric limitations imposed by the relatively short length of laminarin molecules.

We further agree that direct evaluation of branching effects would ideally require comparison with structurally defined linear β-(1→3)-glucans. However, high-molecular-weight unbranched β-(1→3)-glucans, such as curdlan, are generally poorly soluble in aqueous solution, precluding their use in the solution-phase analyses employed in this study. Short-chain linear β-(1→3)-glucan oligomers, although water-soluble, fall below the minimum chain length (DP ≥ 10–11) required for effective Dectin-1 engagement (Ref. 11). Structurally defined linear β-(1→3)-glucans of sufficient length for receptor engagement are therefore not currently applicable to the experimental approaches used here. We have discussed this limitation in the revised manuscript (Page 13, Lines 419-423), and clarified that determining the independent contributions of chain length and branching using structurally defined glucans remains an important future direction that will be essential for establishing how glucan architecture governs Dectin-1 assembly and signaling.

6) Finally, the article would be much stronger with some attempts to propose a 3D model of the complex. The authors state that a helix model of laminarin has been used for rationalizing the AFM images, but the model is not shown or validated. The structure of laminarin is still controversial (single or triple helix) and this is not discussed.

We thank the reviewer for this important suggestion. We agree that the original manuscript

did not sufficiently describe the structural model used for AFM interpretation or discuss the uncertainty surrounding the higher-order structure of laminarin.

To address this point, we have added the structural models used for AFM simulations to the revised Supplementary Information (Fig. S2C,D) and expanded the corresponding Results and Discussion sections (Pages 9-10, Lines 301-308). Simulated AFM images generated from both single-helical and triple-helical β-(1→3)-glucan models were compared with the experimental AFM images. The single-helical model produced pronounced periodic surface features that were not apparent in the experimental data, whereas the triple-helical model more closely reproduced both the observed rod-like morphology and the measured height (~1.3 nm).

We have also expanded the Discussion to acknowledge that the higher-order structure of laminarin remains incompletely resolved and may depend on molecular weight, branching pattern, concentration, and solvent conditions (Page 10, Lines 309-315). We have therefore avoided claiming definitive proof of a triple-helical structure and instead conclude that the AFM observations are more consistent with, and probably represent, a multi-chain helical assembly—potentially a triple helix—under the experimental conditions that we used.

With respect to a 3D structural model of the Dectin-1–laminarin complex, we agree that such a model would be highly valuable. However, we believe that the currently available experimental constraints are insufficient to construct a reliable model. Although the crystal structure of the Dectin-1 carbohydrate-recognition domain is available, the present study provides only the overall 3:3 stoichiometry, the approximate ligand-binding region centered around Trp221 and His223 (Ref. 12), and ligand-dependent receptor proximity inferred from chemical crosslinking. These constraints are insufficient to uniquely define the relative arrangement of the receptor molecules and glucan chain(s) within the complex, a limitation that has now been explicitly mentioned in the revised manuscript (Page 13, Lines 430-433).

We have therefore chosen not to include a speculative 3D model in the revised manuscript and instead focus on the experimentally established stoichiometric, biophysical, and dynamic properties of the assembly. In lieu of an atomic model, Fig. 11 has been revised to provide a schematic mechanistic framework summarizing the experimentally observed stoichiometry and cooperative assembly behavior (Pages 13-14, Lines 441-462; Page 24, Lines 788-800). We explicitly note that this representation is intended as a conceptual model rather than a validated 3D structure. Determining the architecture of the Dectin-1–β-glucan complex remains an important goal for future investigations.

Reviewer #3 (Remarks to the Author):

1) This manuscript focuses on the characterization of the interaction between the Dectin-1,

a lectin involved in innate immunity, and laminarin, a beta-glucan polysaccharide, being beta-glucans the known natural ligands of this lectin. The basis for this interaction are currently not fully understood.

The authors use three different techniques to obtain different types of information. The application of these techniques to this system for the first time (to the best of my knowledge) provides new data on this complex interaction, allowing the authors to propose a novel interaction model. The most significant findings is that the binding imply complexes of stoichiometry 3:3, that there is a preferred laminarin length for the binding, and the existence of positive cooperativity. In my opinion these conclusions are novel, and they will be of interest to the community.

We thank the reviewer for their positive evaluation of our work. We are pleased that the reviewer recognizes the novelty of the 3:3 Dectin-1–laminarin stoichiometry, the identification of a preferred laminarin chain-length window, and the demonstration of positive cooperativity. We appreciate the reviewer's assessment that the combination of native MS, FIDA, and HS-AFM provides new mechanistic insight into Dectin-1–β-glucan recognition and will be of interest to the glycobiology and innate immunity communities.

2) A couple of issues are not clear:

In the MS analysis of laminarin: in Figure 3A it is said that deconvolution of the positive ion mode spectrum was not possible due to overlapping charge states, and it is actually not shown (contrary to the negative ion mode spectrum), while in the manuscript (page 5, lines 144-147) it is stated that “the positive-ion spectrum showed a similar distribution, with a DP of 22-” where this analysis come from if deconvolution is not possible?

We thank the reviewer for pointing out this ambiguity. We agree that the original wording was insufficiently precise and might be interpreted as implying that the positive-ion spectrum was successfully deconvoluted. This was not the case.

Reliable deconvolution of the positive-ion spectrum was not possible because of extensive charge-state overlap, but we were still able to manually assign the underlying charge-state series directly from the raw spectrum. Using these charge-state assignments, we estimated the corresponding molecular-mass distribution and confirmed that it was consistent with the DP 22–44 distribution independently obtained from the deconvoluted negative-ion spectrum.

To avoid confusion, we have revised the manuscript to clearly distinguish between deconvolution-based analysis (negative-ion mode) and charge-state assignment from the raw positive-ion spectrum (Page 5, Lines 137-140).

3) In the high-speed AFM, the interpretation of the authors is that laminarin form a helix, based on a simulated AFM from a model structure of a laminarin helix. How was this structural model generated? Please provide more data: is this a single helix? Laminarin has been proposed to oligomerize, is this taken into account here?

We thank the reviewer for this important comment. We acknowledge that the description of the laminarin structural model was insufficient in the original manuscript. The laminarin model used for AFM simulation was based on a previously reported triple-helical β-(1→3)-glucan structure; AFM images were generated by collision simulation and compared with the experimentally observed AFM images of laminarin adsorbed on mica.

To address the reviewer's concern regarding helix stoichiometry, we have additionally generated simulated AFM images from a single-chain helical β-(1→3)-glucan model and included the results in the revised Supplementary Information (Fig. S2D). The simulated single-helix model produced pronounced periodic surface features that were not observed experimentally. In contrast, the simulated triple-helix model yielded a smoother rod-like morphology, as well as an apparent height that closely matched the experimental AFM data.

We have therefore revised the manuscript to clarify that the AFM observations are more consistent with a multi-chain helical assembly than with an isolated single-chain helix. We have also emphasized that HS-AFM alone cannot definitively distinguish all possible glucan conformations. Accordingly, we have moderated our interpretation and now state that the observed laminarin structures are more consistent with a multi-chain helical assembly—potentially a triple helix—under the experimental conditions used in the study. In addition, the revised manuscript and Supplementary Information now include a description of model generation and the comparison between single- and triple-helix simulations (Pages 9-10, Lines 301-308 and Fig. S2D legend).

Point-by-point reply

Reviewers' comments:

Reviewer #1 (Remarks to the Author):

I am satisfied with the responses and revisions the authors made to critiques, and I am in support of publication at this time.

Reviewer #2 (Remarks to the Author):

The authors provided extensive and well argumented answers to the reviewer questions. The performed new experiments and modified the manuscript has requested.

Reviewer #3 (Remarks to the Author):

The authors have satisfactorily addressed my two concerns. The MS data interpretation has been further clarified (this is also now included in the manuscript). Additionally, simulated AFM images for single-chain glucans have been included, which rule out their major presence, and reinforce the proposed multi-chain helical assembly, while at the same time moderating the interpretation of the Dectin-1 recognised glucan assembly.

We thank all three reviewers for their positive evaluation of our revised manuscript and for supporting its publication. We are pleased that the revisions and new experimental data were found satisfactory.
