# Supplementary material for: Chain-length-selective cooperative assembly underlies β-glucan recognition by Dectin-1
Source: Commun Chem. 2026 Nov 10;9:332. doi: 10.1038/s42004-026-02217-x (PMC13751592; doi:10.1038/s42004-026-02217-x)
Supplement: Supplementary file 2 [file 42004_2026_2217_MOESM2_ESM.pdf]

**Supplementary Information**

# Chain-length–selective cooperative assembly underlies β-glucan recognition by Dectin-1

Noriyoshi Manabe[1], Atsuji Kodama[2], Tokito Osaki[1], Hanako Chiba[1], Yuki Yamaguchi[3], Feng-Yueh Chan[4], Koichi Kato[2], Takayuki Uchihashi[2,4,5,6], Susumu Uchiyama[2,3], Yoshiki Yamaguchi[1,*]

*[1]Division of Structural Glycobiology, Institute of Molecular Biomembrane and Glycobiology, Tohoku Medical and Pharmaceutical University, Sendai, Miyagi 981-8558, Japan; [2]Exploratory Research Center on Life and Living Systems (ExCELLS), National Institutes of Natural Sciences, 5-1 Myodaiji, Okazaki, Aichi 444-8787, Japan; [3]Graduate School of Engineering, The University of Osaka, 2-1 Yamadaoka, Suita, Osaka 565-0871, Japan; [4]Graduate School of Sciences, Nagoya University, Furocho, Chikusa, Nagoya, Aichi 464-8602, Japan; [5]Quantum-Based Frontier Research Hub for Industry Development (Q-BReD), Nagoya University, Nagoya 464-8601, Aichi, Japan; [6]Institute for Glyco-core Research (iGCORE), Nagoya University, 464-8602 Nagoya, Japan*

**Figures: S1-S4**
**Movies: S1-S5**

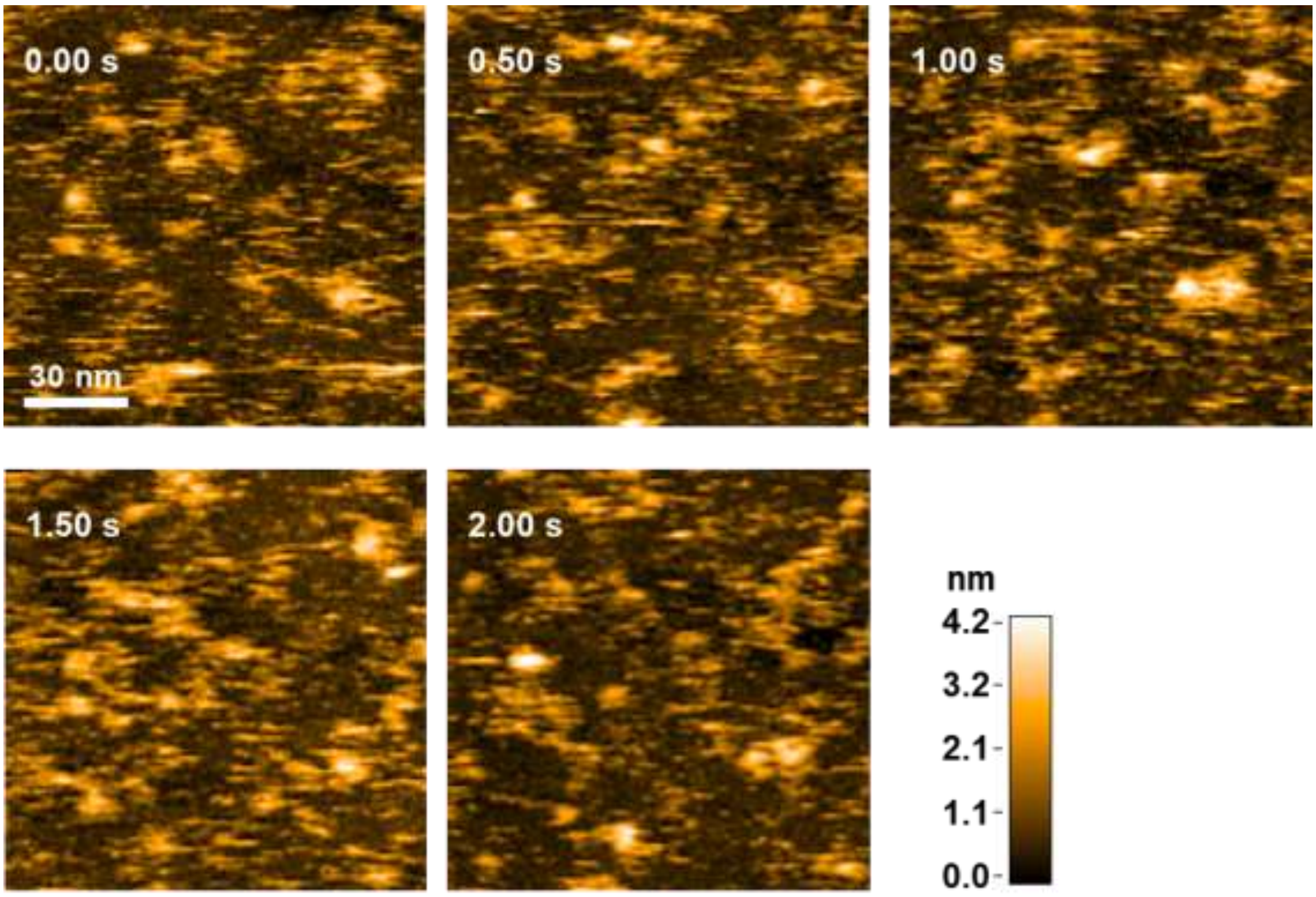


**Fig. S1. HS-AFM images of Dectin-1 alone on mica.** HS-AFM images of Dectin-1 (1 μM) deposited on mica in PBS (pH 7.4). Dectin-1 molecules diffuse rapidly across the mica surface, preventing clear identification of their shape. A few stationary bright spots are also observed. Imaging speed, 0.5 s/frame.

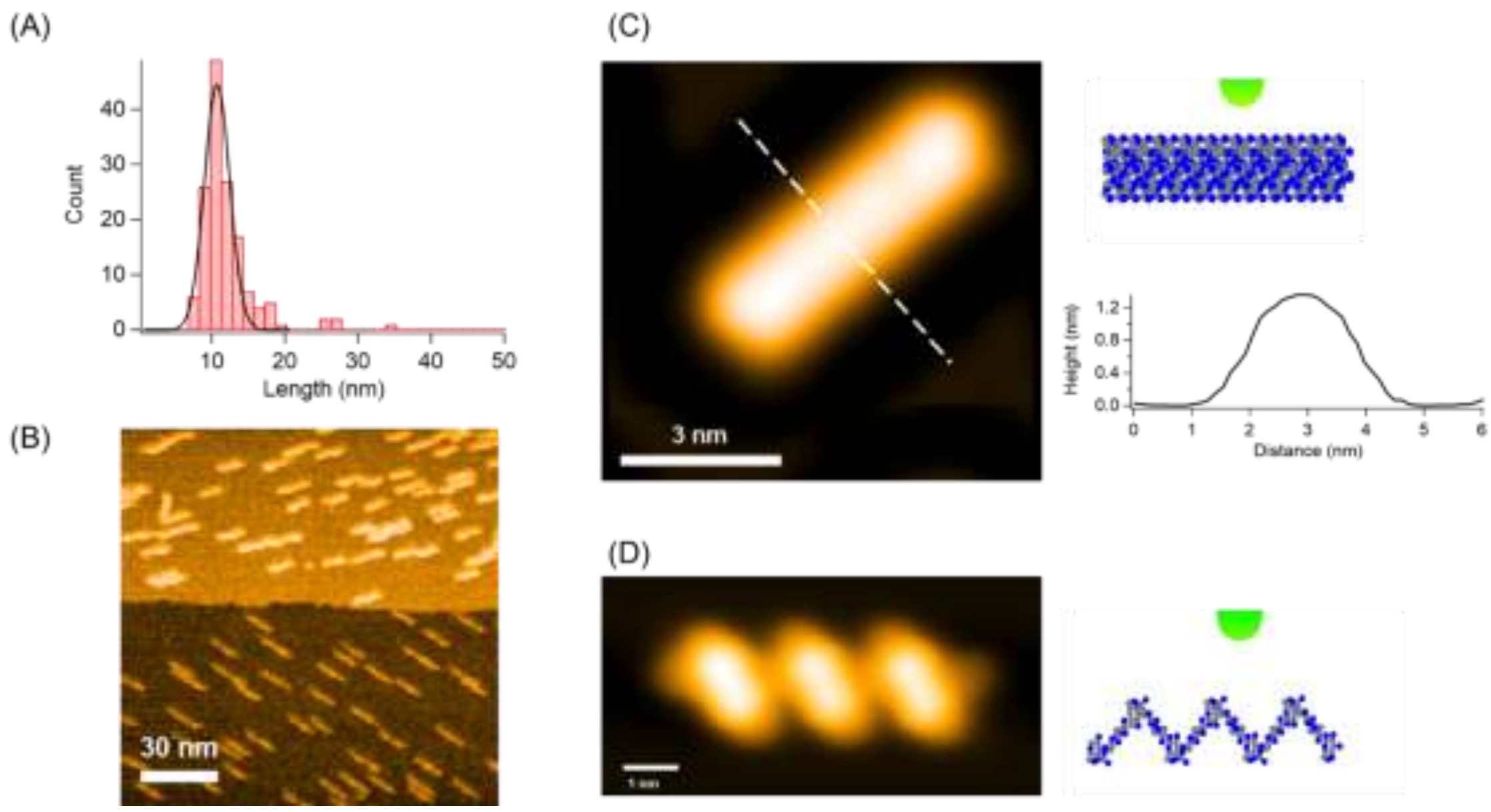


**Fig. S2. Structural characterization of laminarin adsorbed on mica.** (**A**) Histogram of rod lengths of laminarin (100 µM) adsorbed on mica ($n = 147$). Unbroken line represents a Gaussian fit (peak, 9.96 ± 0.05 nm). (**B**) HS-AFM image of laminarin rod-like structures near a mica step edge. The orientation of the rods rotates by ~60° across the step, indicating epitaxial adsorption in register with the crystalline mica lattice. (**C**) Simulated AFM image generated from a laminarin triple helix model structure using a collision simulation (tip radius, 0.5 nm; cone angle, 10°). The simulated height of the helix is ~1.2 nm, consistent with the experimentally observed rod height. (**D**) Simulated AFM image generated from a laminarin single helix model structure using a collision simulation (tip radius, 0.5 nm; cone angle, 10°). Comparison of the experimental AFM image with the simulated images shown in (**C**) and (**D**) suggests that the observed morphology is consistent more with a triple-helical assembly than with an extended single-chain conformation.

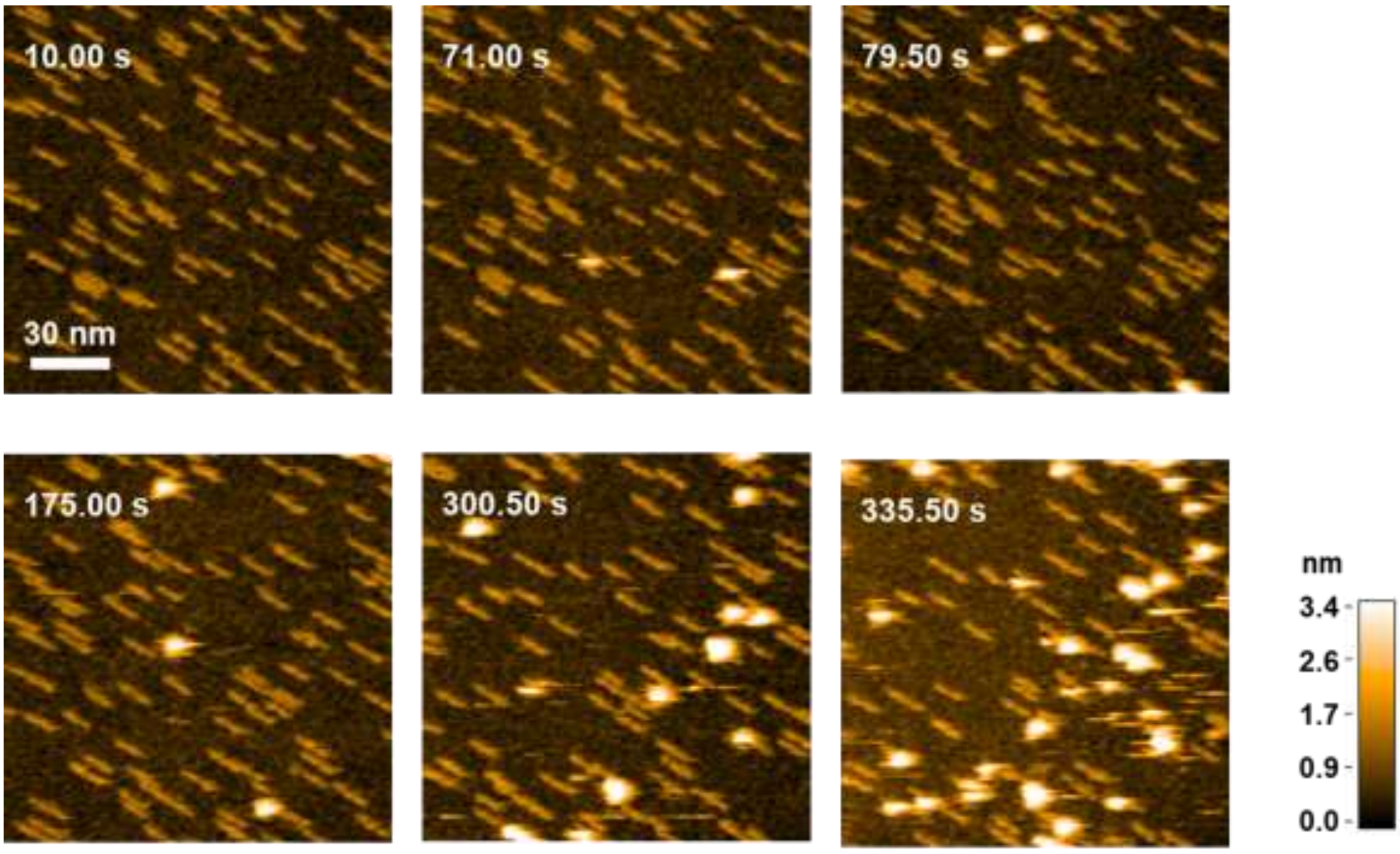


**Fig. S3. Time-lapse HS-AFM images of Dectin-1–laminarin complex formation.** Sequential HS-AFM images recorded after addition of Dectin-1 (0.3 μM) to laminarin (100 μM) pre-adsorbed on mica in PBS (pH 7.4) (corresponding to **Movie S2**). Gradual adsorption of Dectin-1 onto laminarin is observed over time. The indicated times correspond to time elapsed after Dectin-1 addition. Imaging speed, 0.5 s/frame.

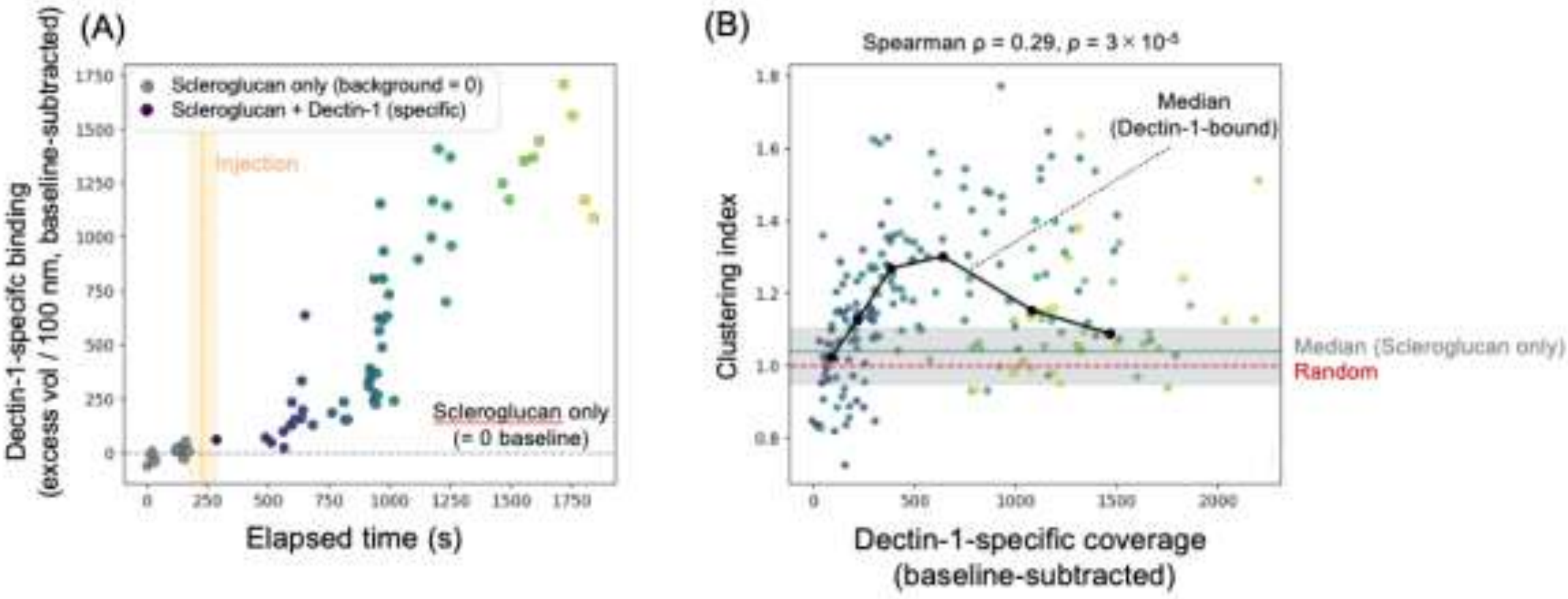


**Fig. S4. High-speed AFM analysis of Dectin-1–scleroglucan complex formation.** (**A**) Time-dependent association of Dectin-1 with individual scleroglucan chains. Horizontal axis indicates elapsed time; vertical axis indicates background-corrected Dectin-1–specific excess volume per 100 nm of chain length. Protein-derived signals were obtained by subtracting the chain-only baseline. The median trajectory is shown together with individual chain traces to illustrate both the overall trend and chain-to-chain variability. (**B**) Relationship between Dectin-1 coverage and clustering on scleroglucan chains. Horizontal axis represents background-corrected Dectin-1 coverage, expressed as excess volume per 100 nm of chain length; vertical axis shows the clustering index, which quantifies the degree of spatial localization relative to a random distribution. Individual measurements are displayed as colored points, with color indicating elapsed time. The median trend is shown to illustrate the increase in clustering with increasing Dectin-1 coverage.

**Movie S1. High-speed AFM observation of Dectin-1 alone on mica.**
HS-AFM movie of Dectin-1 (1 μM) deposited on mica in PBS (pH 7.4). Most Dectin-1 molecules diffuse rapidly across the mica surface, precluding clear identification of their individual molecular shapes. Imaging speed, 0.5 s/frame.

**Movie S2. Real-time formation of Dectin-1–laminarin complexes.**
HS-AFM movie recorded after the addition of Dectin-1 (final concentration 0.3 μM) to laminarin (100 μM) pre-adsorbed on mica in PBS (pH 7.4). The video shows the gradual adsorption and accumulation of Dectin-1 molecules onto the rod-like laminarin structures over time. Imaging speed, 0.5 s/frame.

**Movie S3. Dynamic association and dissociation of Dectin-1 on laminarin.**
High-resolution HS-AFM movie showing Dectin-1 (0.3 μM) repeatedly binding to and dissociating from laminarin chains (100 μM). The movie captures the dynamic behavior of Dectin-1, illustrating that while individual binding events can be transient, the presence of neighboring molecules on the same chain leads to enhanced stability of the complex. Imaging speed, 0.15 s/frame.

**Movie S4. Time-resolved HS-AFM observation of Dectin-1 accumulation on scleroglucan.**
HS-AFM movie recorded after the addition of Dectin-1 to scleroglucan pre-adsorbed on freshly cleaved mica in PBS (pH 7.4). Individual Dectin-1 molecules are seen on the scleroglucan chains shortly after addition and progressively accumulate over the observation period, consistent with the time-dependent increase in Dectin-1–specific binding shown in Fig. S4A. Intervals without substantial change were omitted for clarity, as indicated by fade-out/fade-in transitions. Dectin-1 final concentration, 0.7 μM; imaging speed, 0.2 s/frame.

**Movie S5. HS-AFM observation of Dectin-1 clustering on a single scleroglucan chain.**
Close-up time-lapse HS-AFM movie of an individual scleroglucan chain recorded in PBS (pH 7.4) at a late stage after Dectin-1 addition, when receptor occupancy is high. Numerous Dectin-1 molecules are densely clustered in patches along the chain rather than distributed uniformly, illustrating the cooperative clustering quantified in Fig. 9. Dectin-1 final concentration, 0.7 μM; imaging speed, 0.15 s/frame.
