# Supplementary material for: Chain-length-selective cooperative assembly underlies β-glucan recognition by Dectin-1
Source: Commun Chem. 2026 Nov 10;9:332. doi: 10.1038/s42004-026-02217-x (PMC13751592; doi:10.1038/s42004-026-02217-x)
Supplement: Supplementary file 3 [file 42004_2026_2217_MOESM3_ESM.pdf]

Description of Additional Supplementary Files:

File: Supplementary Movie S1

Description: High-speed AFM observation of Dectin-1 alone on mica.

HS-AFM movie of Dectin-1 (1 μM) deposited on mica in PBS (pH 7.4). Most Dectin-1 molecules diffuse rapidly across the mica surface, precluding clear identification of their individual molecular shapes. Imaging speed, 0.5 s/frame.

File: Supplementary Movie S2

Description: Real-time formation of Dectin-1–laminarin complexes.

HS-AFM movie recorded after the addition of Dectin-1 (final concentration 0.3 μM) to laminarin (100 μM) pre-adsorbed on mica in PBS (pH 7.4). The video shows the gradual adsorption and accumulation of Dectin-1 molecules onto the rod-like laminarin structures over time. Imaging speed, 0.5 s/frame.

File: Supplementary Movie S3

Description: Dynamic association and dissociation of Dectin-1 on laminarin.

High-resolution HS-AFM movie showing Dectin-1 (0.3 μM) repeatedly binding to and dissociating from laminarin chains (100 μM). The movie captures the dynamic behavior of Dectin-1, illustrating that while individual binding events can be transient, the presence of neighboring molecules on the same chain leads to enhanced stability of the complex. Imaging speed, 0.15 s/frame.

File: Supplementary Movie S4

Description: Time-resolved HS-AFM observation of Dectin-1 accumulation on scleroglucan.

HS-AFM movie recorded after the addition of Dectin-1 to scleroglucan pre-adsorbed on freshly cleaved mica in PBS (pH 7.4). Individual Dectin-1 molecules are seen on the scleroglucan chains shortly after addition and progressively accumulate over the observation period, consistent with the time-dependent increase in Dectin-1–specific binding shown in Fig. S4A. Intervals without

substantial change were omitted for clarity, as indicated by fade-out/fade-in transitions. Dectin-1 final concentration, 0.7 μM; imaging speed, 0.2 s/frame.

File: Supplementary Movie S5

Description: HS-AFM observation of Dectin-1 clustering on a single scleroglucan chain.

Close-up time-lapse HS-AFM movie of an individual scleroglucan chain recorded in PBS (pH 7.4) at a late stage after Dectin-1 addition, when receptor occupancy is high. Numerous Dectin-1 molecules are densely clustered in patches along the chain rather than distributed uniformly, illustrating the cooperative clustering quantified in Fig. 9. Dectin-1 final concentration, 0.7 μM; imaging speed, 0.15 s/frame

File: Supplementary Data 1

Description: The source data for Figure 6.

File: Supplementary Data 2

Description: The source data for Figure 7.

File: Supplementary Data 3

Description: The source data for Figure 10.
